# Transcriptomic Maps of Colorectal Liver Metastasis: Machine Learning of Gene Activation Patterns and Epigenetic Trajectories in Support of Precision Medicine

**DOI:** 10.3390/cancers15153835

**Published:** 2023-07-28

**Authors:** Ohanes Ashekyan, Nerses Shahbazyan, Yeva Bareghamyan, Anna Kudryavzeva, Daria Mandel, Maria Schmidt, Henry Loeffler-Wirth, Mohamed Uduman, Dhan Chand, Dennis Underwood, Garo Armen, Arsen Arakelyan, Lilit Nersisyan, Hans Binder

**Affiliations:** 1 Armenian Bioinformatics Institute, 3/6 Nelson Stepanyan Str., Yerevan 0062, Armenia; ohanes.ashekyan@abi.am (O.A.); nerses.shahbazyan@abi.am (N.S.); yeva.bareghamyan@abi.am (Y.B.); anyakudryavceva804@gmail.com (A.K.); daria.laricheva@abi.am (D.M.); lilit.nersisyan@abi.am (L.N.); 2 IZBI, Interdisciplinary Centre for Bioinformatics, Universität Leipzig, Härtelstr. 16–18, 04107 Leipzig, Germany; schmidt@izbi.uni-leipzig.de (M.S.); wirth@izbi.uni-leipzig.de (H.L.-W.); 3 Agenus Inc., 3 Forbes Road, Lexington, MA 7305, USA; mohamed.uduman@agenusbio.com (M.U.); dhan.chand@agenusbio.com (D.C.); dennis.underwood@agenusbio.com (D.U.); armen@agenusbio.com (G.A.); 4 Institute of Molecular Biology of the National Academy of Sciences of the Republic of Armenia, 7 Has-Ratyan Str., Yerevan 0014, Armenia; arsen.arakelyan@abi.am

**Keywords:** genomics analysis of liver metastases, gene expression, molecular mechanisms, tumor heterogeneity, prognostic score, treatment resistance, self-organizing map machine learning

## Abstract

**Simple Summary:**

Liver metastasis is a significant factor contributing to mortality associated with colorectal cancer. Establishing the biological mechanisms of metastasis is crucial for refining diagnostics and identifying therapeutic windows for interventions. Currently, little is known of the processes that govern the development of liver metastases, the role of the tumor microenvironment, the role of epigenetics, and potential treatment-induced shaping effects. Machine learning-based bioinformatics has provided an important methodical option to decipher fine-granular details of the transcriptomic landscape of tumor heterogeneity and the underlying molecular mechanisms. Our molecular portrayal method has potential implications for treatment decisions, which may require personalized diagnostics.

**Abstract:**

The molecular mechanisms of the liver metastasis of colorectal cancer (CRLM) remain poorly understood. Here, we applied machine learning and bioinformatics trajectory inference to analyze a gene expression dataset of CRLM. We studied the co-regulation patterns at the gene level, the potential paths of tumor development, their functional context, and their prognostic relevance. Our analysis confirmed the subtyping of five liver metastasis subtypes (LMS). We provide gene-marker signatures for each LMS, and a comprehensive functional characterization that considers both the hallmarks of cancer and the tumor microenvironment. The ordering of CRLMs along a pseudotime-tree revealed a continuous shift in expression programs, suggesting a developmental relationship between the subtypes. Notably, trajectory inference and personalized analysis discovered a range of epigenetic states that shape and guide metastasis progression. By constructing prognostic maps that divided the expression landscape into regions associated with favorable and unfavorable prognoses, we derived a prognostic expression score. This was associated with critical processes such as epithelial–mesenchymal transition, treatment resistance, and immune evasion. These factors were associated with responses to neoadjuvant treatment and the formation of an immuno-suppressive, mesenchymal state. Our machine learning-based molecular profiling provides an in-depth characterization of CRLM heterogeneity with possible implications for treatment and personalized diagnostics.

## 1. Introduction

Liver metastasis, linked with poor prognosis, is a common occurrence in various cancers, including colorectal cancer (CRC), pancreatic cancer, breast cancer, melanoma, and lung cancer. In CRC, the liver is the primary site of metastasis owing to the anatomical and vascular connections between the colorectal regions and the liver [1]. Such metastases pose a significant challenge for clinical intervention and represent a major cause of CRC-related mortality. However, the molecular mechanisms that govern the molecular heterogeneity and tumor development in liver metastasis remain poorly understood.

The tumor microenvironment (TME) significantly impacts the pathophysiology of cancer cells metastasizing to the liver. It includes liver sinusoidal endothelial cells, Kupffer cells, hepatic stellate cells, and parenchymal hepatocytes, as well as infiltrating stromal and immune cells [2]. Interactions with the TME facilitate cancer cells to overcome the tumor stroma, settle, and to colonize. Interestingly, colorectal cancer liver metastases (CRLM) show high genetic concordance in key lesions, mutations, and copy number variations (CNV) with primary CRC suggesting the liver microenvironment has a limited influence on the mutation pattern of CRLM cells, which remain largely genetically primed by their primary CRC origin [3]. This correlation of molecular characteristics between primary and metastatic tumors is further supported by the functional and transcriptional studies on CRLM [3,4,5,6,7]. However, genetic lesions and adaptive interactions with the TME, while necessary, are not sufficient for cancer initiation and progression. As a third factor, epigenetic regulation, including chromatin remodeling associated and driven by a large set of histone- and DNA-modifying mechanisms, are essential for cancer clones to acquire the plasticity necessary for adaptive cell fate changes towards evolutionary fitness in an epigenetic landscape [8,9]. Currently, little is known of the evolutionary processes that govern CRLM, the role of epigenetics, and potential treatment-induced shaping effects. Establishing the biological mechanisms of metastasis is crucial for refining diagnostics and identifying therapeutic windows for interventions. 

Molecular subtyping has emerged as an important concept to decipher cancer heterogeneity in both primary and metastatic tumors [10]. While correlations between the molecular subtypes of primary CRC, metastatic propensity, and responses to therapy have been noted [6], it is not clear to what degree these CRC characteristics are maintained after distant spread. Recently, Moosavi et al. developed a metastasis-oriented subtyping framework through the transcriptomic analysis of patients with CRLMs [5]. Their de novo liver metastases subtypes (LMS) recapitulated epithelial-like and mesenchymal-like tumors, with the latter showing a strong immune and stromal component. 

In this study, we leveraged machine learning-based bioinformatics to analyze the transcriptomes of 283 CRLM [5], to characterize the molecular landscape with single-tumor resolution, and to deduce cues for CRLM development under neoadjuvant treatment. We further evaluated and extended the LMS framework, and identified subtype-specific gene signatures, explored their functional relevance to cancer progression, and suggest possible developmental paths under epigenetic control using pseudotime inference through in-depth transcriptomic analysis. Our approach utilizes a high-resolution molecular cartography and portrayal method previously applied to various cancer types [11,12,13,14], treatment resistance [15], and modes of epigenetic regulation [16,17]. In parallel, we have established, and made available, an interactive web platform for more detailed insights into our analyses. Through the personalized portrayal of gene expression data and the creation of prognostic maps for CRLM, we open new avenues for personalized diagnostics and treatment decision-making.

## 2. Materials and Methods

### 2.1. Gene Expression Data of Colon Liver Metastases (CRLM)

Microarray-based gene expression data (GeneChip Human Transcriptome Array 2.0; HTA2.0) of 283 hepatic resections of liver metastases (CRLM) of primary colorectal (CRC) tumors were taken from GEO-database under accession numbers GSE159216 (see also [5]) covering the expression of, in total, more than 67,000 probe sets. We used the classification of CRLM-samples into five liver metastasis subtypes (LMS1-LMS5) and an “unclassified” group, all taken from the original paper [5]. The latter class showed an expression signature of healthy liver tissue and therefore it was assigned as liver-like (LIV, see Section 3). We used preprocessed gene expression data, as described in [5].

### 2.2. SOM Portrayal and Downstream Functional Analysis

The gene expression data were transformed to log10-scale, then quantile normalized and centralized (with respect to the mean log-expression of the gene averaged over all CRLM studied). Throughout the paper, we use the terms “over-“ and “under-“expression for positive and negative values (larger/smaller than the mean value, respectively). Expression profiles denote the vector of expression data of each transcript across all CRLM-samples. Expression profiles were clustered using self-organizing map (SOM) machine learning. This method transforms the ~67,000 transcript-centric profiles into 2500 metagene profiles of reduced dimensionality and visualizes their expression in each CRLM sample as two-dimensional quadratic images with 50 × 50 metagene resolution [18]. Metagenes were colored according to their expression for each CRLM from dark blue (low expression) to maroon (high expression), thereby providing a portrait of their gene expression state. An alternative “coastline” color scale was applied to better resolve subtle expression changes for over (red) and under (blue) expression regions. The SOM clustered similar profiles of co-expressed genes together, appearing as spot-like regions in the portraits [18]. Subtype-specific mean portraits were generated by averaging the metagene landscapes of all CRLM belonging to the same class. Difference portraits between subtypes were calculated as the differences between the respective mean portraits. Details of SOM training and parametrization have been previously described [18,19]. Bioinformatics downstream analyses, including class discovery and sample diversity analysis, feature selection from metagenes and spots, and biological function-mining using gene sets analysis, were performed as described in [18,20]. All downstream methods were implemented in the R-package ‘oposSOM’ [21] and applied for analysis. Gene sets implemented specifically for this study are provided in Supplementary Table S2. Single-cell RNAseq data from Che et al. [22] (GSE178318) on CRLM were re-analyzed to extract cell-specific marker genes. 

### 2.3. Prognostic Maps

For prognostic maps, the hazard ratio (HR) relative to the mean overall survival (OS) of all patients was calculated for each metagene-pixel on the map. This was done by selecting patients with a centralized metagene expression greater than zero or one standard deviation and coloring the pixel in an HR-scale from red (indicating inferior prognosis) to blue, as described in [11].

### 2.4. oposSOM Browser of Livermet-Transcriptomes

The results of transcriptome analyses of CRLM presented in this publication can be interactively explored for further details using the oposSOM browser [23], available via the IZBI-portal (www.izbi.de, accessed on 27 July 2023; https://apps.health-atlas.de/opossom-browser/?dataset=15, accessed on 27 July 2023). For more information refer to the data availability statement below and Supplementary Figure S1.

## 3. Results

### 3.1. SOM Portrayal Deciphers Liver Metastases Subtypes (LMS) 

**Figure 1:**
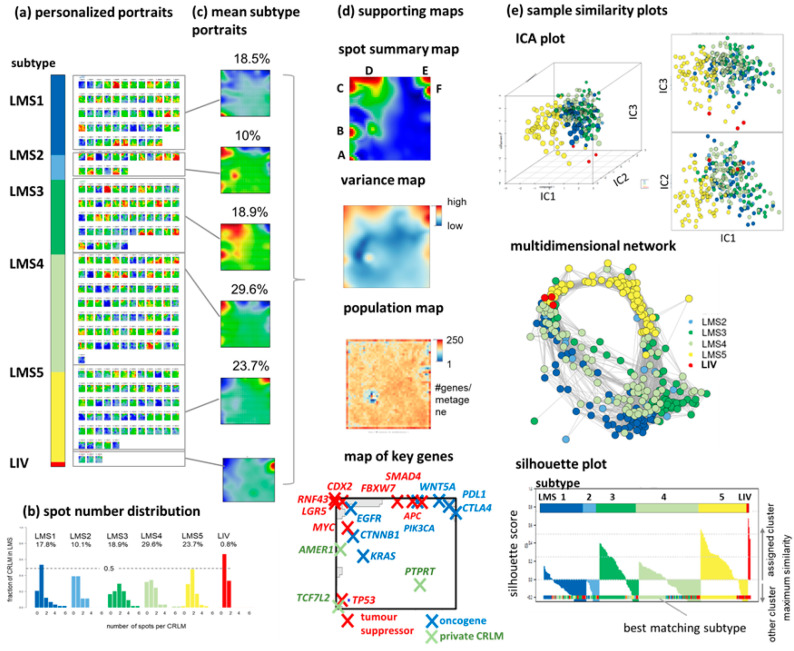
SOM portrayal of LMS subtypes. (**a**) Individual gene expression SOM portraits of the 283 liver metastases (CRLM) samples taken from [5] (see Supplementary Figure S2 for an enlarged portrait gallery). (**b**) Mean portraits of each of the subtypes LMS 1-5 and LIV (CRLM strongly contaminated with liver tissue). N assigns the respective sample size. (**c**) Distribution of spot numbers in the individual portraits of each subtype. (**d**) Supporting maps summarize the major overexpression spots containing highly variant genes across all the portraits (assigned with capital letters A–F; the variance of gene expression across the map; the color-coded distribution of the number of genes in each of the 2500 pixels of the map called metagenes; and the locations of key CRC- and CRLM-related genes on the SOM portraits. (**e**) Sample similarity relations are visualized using Independent Component Analysis (ICA) and a multidimensional network. The silhouette plot estimates the similarity of each sample to its own subtype cluster through silhouette scores, and indicates the most similar subtype with a color-code bar below.

The self-organizing maps (SOM) algorithm was applied to transcriptomic data, generating individual SOM portraits of each of the 283 CRLM samples (Figure 1a and Figure S2). These portraits were group-sorted according to their previous classification [5] into five subtypes LMS1–LMS5 and one “unclassified” group, LIV, which strongly resembled the transcriptional characteristics of healthy liver tissue. Mean portraits of each subtype indicate the specific expression patterns of spot-like clusters of overexpressed and under-expressed genes, using a red-to-blue color scale, respectively (Figure 1b). 

The distribution of spot numbers reveals that the personalized portraits most frequently express one to three such spot-clusters (Figure 1c). LMS3 and LMS4 display the broadest distributions of spot numbers and, consequently, the most heterogeneous expression patterns. The most variable genes across the datasets are located in six major spot modules of the genes coregulated across the subtypes assigned with capital letters A–F (see the summary and variance maps Figure 1d). Each map is composed of 2500 pixels, also referred to as metagenes, each containing up to 200 genes (see the population map in Figure 1d). Key genes harboring somatic mutations and/or copy number variations (CNV) in CRC and CRLM [24,25,26] are located in or near the spots (Figure 1d, part below), which includes canonical core CRC drivers (combinations of *APC*, *KRAS*, *TP53*, or *SMAD4*), which can combine with one additional candidate metastasis driver (*TCF7L2*, *AMER1*, or *PTPRT*) [27]. 

Similarity plots of the CRLM using independent component analysis (ICA, Figure 1e) and a multidimensional network presentation (Figure 1e) reveal the intermixing of the CRLM of nearly all LMS, except for LMS5 and LIV (see also Supplementary Figure S3). This is also reflected in the silhouette plot that compares intra- versus inter-cluster similarities of the samples, where, except LMS5 and LIV, a large fraction of the CRLM show high preferences for inter-cluster similarities. This, overall, confirms the heterogeneous transcriptional patterns of CRLM and their stratification into five LMS and a LIV group enriched in healthy liver tissue.

### 3.2. LMS Are Governed by Six Major Modules of Co-Regulated Genes

**Figure 2:**
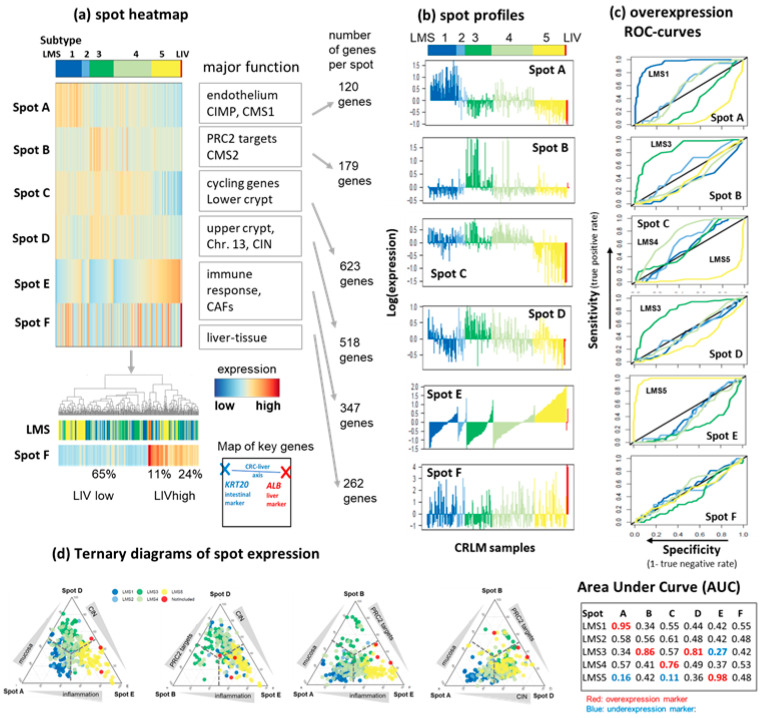
Expression profiles of the spot-modules. (**a**) The heatmap shows sample-specific expression of each spot, grouped by subtypes. Hierarchical clustering of samples according to spot F expression is shown below along with the expression of the spot F that reflects lower and higher liver cell content in LIVlow and LIVhigh, respectively. The percentage of samples classified into high and low spot F expression profiles is shown below. Gene set analysis associates spot genes with biological function (see Supplementary Table S1 and text). (**b**) Barplots of spot expression profiles are sorted with increasing expression of spot E in each subtype to better visualize co-expression of the other spots with inflammatory response. (**c**) Receiver operating characteristic curves (ROC) of subtype classification power of each spot expression. ROC curves below the diagonal line (AUC < 0.5) mean that underexpression (low sensitivity) is a better predictor than overexpression (high sensitivity). AUC values are listed in the table below. (**d**) Ternary diagrams combine expression of selected spot tuples.

The spots A–F taken from the mean LMS portraits represent modules containing about 200 (spot A) to 623 (spot C) co-expressed genes (Figure 2a, lists of the spot genes are provided in Supplementary Table S2). Their expression profiles reveal the overexpression of spot A in LMS1, spots B and D in LMS3, spot C in LMS4, spot E in LMS5, and the under-expression of spots A and C in LMS5 (Figure 2b and Figure S3). Their functional context was established by gene set analysis using an array of gene sets implemented in oposSOM (see Figure 2a, Supplementary Table S1 and next subsection). The spot profiles show the expression of the spot modules as bar plots, where the samples within each subtype are ordered with the increasing expression values of the spot E that shows inflammatory characteristics (Figure 2b). The negative slope of the expression values of spot D, associated with upper crypt genes and chromosomal instability (CIN) anticorrelates with spot E. Conversely, the expression of spot A (epithelium) positively and negatively correlates with the inflammatory characteristics, respectively, suggesting an overlay of various immune-related functions in LMS1 and LMS5. Overall, the distinct spot profile indicates complex co-expression patterns governing a multitude of cellular functions, which are addressed below.

Spot F exhibits the expression characteristics of healthy liver tissue, with high values in the LIV group and in about 35% of the CRLM samples across all LMS, suggesting contamination with liver tissue (Figure 2b, part below). These samples are prone to LMS misclassification, as most of them show a negative silhouette score and preference for the LIV-group (Figure 1d). Liver (*ALB*) and intestine gene marker genes (*KRT20*) [5] are located in spots F and C, respectively, defining a major transcriptomic axis between liver and CRLM.

The receiver operating characteristic (ROC) curves indicate a high classification power for the overexpression of spot A for LMS1 (AUC = 0.95), of spot B (0.86) and D (0.81) for LMS3, of spot C for LMS4 (0.76), and of spot E for LMS5 (0.98) (Figure 2c). LMS2 exhibits mixed characteristics, combining moderate AUCs for spots A, B, and C. Notably, all the nine genes of the mini classifier distinguishing between LMS1 and other subtypes [5] are located in spot A. Spot C (cycling genes) is a specific under-expression marker for LMS5 (AUC =0.11), implying that cell-cycle activity in LMS5 is distinctly reduced compared with the other LMS. ROCs of spot F reflect no preference for any LMS, thus supporting the view that contaminations with healthy liver tissue are distributed over all LMS. 

The combined classification power of these spots is demonstrated using ternary diagrams of their expressions (Figure 2d). A ternary combination of spots A, D, and E provides the best resolution, with LMS1 distributing to the spot A corner, while LMS3 and LMS5 distribute to the D and E corners, respectively, and LMS4 to the middle. LMS2 could not be specifically classified with single spot markers. Other combinations of spots in the ternary diagrams show a depleted population of the B-corner, especially in combination with spots A and D. 

Overall, the six overexpression spots provide transcriptomic marker signatures of four (LMS1, LMS3, LMS4, LMS5) of the five subtypes. Triple combinations of spots A (epithelium), D (CIN), and E (endothelium/mesenchyme and inflammation) best resolve subtypes LMS1, LMS3, LMS 4, and LMS5, respectively. Therefore, the six overexpression spots provide transcriptomic marker signatures of the subtypes where triple combinations of epithelial- (spot A), CIN (spot D), and mesenchymal/inflammation (spot E) best resolve the subtypes LMS1, LMS2-4, and LMS5, respectively.

### 3.3. Functional and TME Context and Relation to Primary CRC Characteristics 

**Figure 3:**
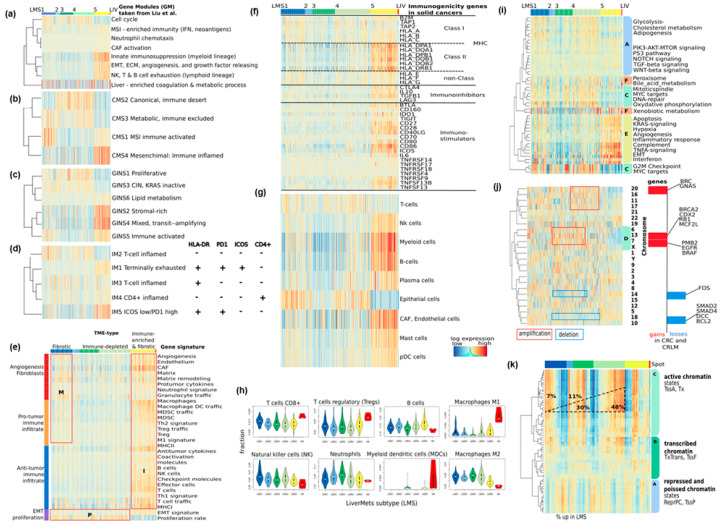
Functional characteristics of CRLM using gene set Z scores (GSZ) profiling in CRLM of various signatures: (**a**) Expression of gene modules (GM) extracted from an independent study [4] confirm LMS and spot clusters in this study. (**b**) Consensus molecular subtypes of CRC (CMS1-4) [24] resemble CRLM expression. (**c**) There is partial concordance between the gene interaction perturbation network subtypes (GINS1-6) signatures [28] and the LMS expression patterns. (**d**) Immune microenvironment types (IM1-5) relate the LMS to specific activation patterns of immune checkpoint inhibitors and *HLA-DRB* [3]. (**e**) Tumor microenvironment (TME) PanCancer signatures taken from [29] assign the LMS to different TME-subtypes. (**f**) Immunogenicity genes in solid cancers were mostly upregulated in LMS5 [30]. (**g**) Cell specific expression markers extracted from a single-cell RNA analysis of CRLM [22] activate in two clusters in LMS1 and LMS5, respectively. (**i**) Hallmark signatures of cancer [36] confirm the functional assignment of LMS and spots. (**j**) Genes on chromosomes show dose response effects of copy number gains (red frame) and losses (blue) typically observed in CRC and CRLM [41]. (**k**) Epigenetic signatures identify two distinct patterns across all LMS, one related to open, actively transcribed genes, the other to repressed and poised ones [42]. (**h**) Cell-deconvolution of CRLM transcriptomics data [43] reveals LMS-specific fractions of selected cell types. Gene sets implemented in this study are given in Supplementary Table S3.

To further characterize the functional context of the CRLM subtypes, we analyzed a variety of gene sets implemented in the oposSOM software package with heatmaps (Figure 3 and Figure S4), with single-set profiles and gene set maps for detailed inspection (Supplementary Figure S5), and spot enrichment. For comparison with a prior independent gene expression study on CRLM, we considered twenty-six gene modules (GM1-26) identified by the authors [4] and computed their GSZ scores in our samples (Figure 3a). The gene sets related to endothelial characteristics, angiogenesis, and innate immunity were upregulated in LMS5, those related to cell cycle—in LMS1-4, those related to inflammation and neoantigens found in microsatellite instable (MSI) CRC in LMS1, and those specific to liver-tissue spread across all LMS. 

Next, we analyzed the expression signatures of primary CRC subtypes in our LMS-stratified CRLM data (Figure 3b–d and Figure S5a). Signatures of four consensus molecular subtypes (CMS1-4) of CRC [24] indicate associations between the subtypes of the primary tumors and of liver metastases, namely CMS1 (immune-activated) with LMS1 (and to a less degree with LMS5), CMS2 (canonical, *WNT*-signaling) with LMS2-4, CMS3 (metabolic) with LMS4 (and partly LMS1), and CMS4 (mesenchymal) with LMS5 (Figure 3b, see also [5]). This indicates the resemblance of transcriptional programs between LMS and CMS subtypes, which is further supported with signatures of another gene-interaction-perturbation-network-based subtyping (GINS1-6) [28] (Figure 3c).

To gain further insight into the immunogenetic properties of LMS, we assessed signatures associated with immune microenvironmental subtypes (IM1-5). These subtypes consider the activation patterns of the *MHCII* receptor *HLA-DR* and of the immune checkpoint inhibitors (ICI) *PD1* and *ICOS* related to T-cell states (Figure 3d) [3]. According to these patterns, the inflamed IM2 type, which resembles LMS1, is characterized by deactivated ICIs. In contrast, the LMS5-resembling IM1 and IM5 exhibit a T-cell-exhausted immune environment. This is regulated by the hypoxia-marker *SLCA1* with inferior survival risk and treatment resistance [3]. 

Next, we considered a series of signatures characterizing the tumor microenvironment (TME, Figure 3e–h). A pan-cancer classification of TME-types [29] shows that LMS1 corresponds to a fibrotic TME-type, LMS2–LMS4 to an immune depleted and proliferative TME-type, and LMS5 to an immune-enriched and fibrotic TME-type (Figure 3e). For a closer look, we mapped immunogenicity-related genes in solid cancers to the CRLM [30] (Figure 3f and Figure S4). Accordingly, the expression of MHC class II, and both immune-inhibitor and -stimulator genes, upregulate in LMS5, while MHC class I shows slight activity in both LMS1 and LMS5. To obtain a more specific picture, we next studied signatures extracted from the single-cell transcriptomics data of CRLM [22] (Figure 3g and Figure S6). It shows that endothelial cell activity in LMS5 is accompanied by upregulated expression from a series of immune cells such as B-, natural killer (NK)-, myeloid, plasma-, and plasmacytoid dendritic (pDC) cells. It is reported that, e.g., pDCs, promote the recruitment of regulatory T cells (Tregs) into the tumor microenvironment, leading to immune suppression and promoting tumor growth [31]. Contrarily, epithelial and T cells are activated in LMS1 and, partly, LMS4. Cell-type deconvolution refines this result in terms of the fraction of immune cells in the TME (Figure 3h) [32]. Indeed, CD8+ T- and NK-cell invasion is most prominent in LMS1, while regulatory T and B cells are enriched in LMS5. Interestingly, the frequencies of tumor-suppressive M1- and of tumor-promoting M2-macrophages are high and low, respectively, which suggests a pivotal role of macrophage polarization in CRLM in analogy to primary CRC [33,34,35]. We found similar activity profiles across the LMS using specific marker sets for M2-like (*SPP1+* and *MRC1 + CCL18*) macrophages identified in CRLM and possibly originating from liver-intrinsic Kupffer cells [7]. 

Finally, we were interested in signatures related to cancer hallmarks and copy number variations along the chromosomes and chromatin states associated with genetics and epigenetics, respectively (Figure 3i–k and Figure S5c–e). Cancer hallmark signatures [36] reveal functional associations in a broader context (Figure 3i), which generally reflect subtype-specific activations of the hallmark programs. For example, the epithelial–mesenchymal transition (EMT) signature is activated in LMS5, while being downregulated in LMS1, corresponding to the mesenchymal and epithelial nature of these subtypes. Gene expression and copy number variations (CNV) are typically linked via a dose–response relationship [37]. We found that the genes on chromosomes 7 and 13, both showing CNV gains in primary CRC and CRLM [38], accumulate in spot D and are upregulated in LMS3 (and, partly LMS4), while the genes on, e.g., chromosome 18 showing CNV-losses in CRC and CRLM, downregulate in these LMS (Figure 3j, see red and blue boxes, respectively). Overall, we observed the systematic over- and under-expression of genes along chromosomes known for their CNV gains and losses in CRC and CRLM: they accumulate in the CIN-related LMS3 (and partly LMS4) and thus govern the genomic regulation of these LMS. 

We were also interested in the footprints of (epi-)genetic effects in the CRLM transcriptomes. Gene sets for different chromatin states in colonic tissue were taken from [39] and mapped to the CRLM transcriptomes (Figure 3k and Figure S5e, see also [40]). Genes with active (TssA) and with repressed and poised (ReprPC and TssP, respectively) promoters were upregulated in a virtually exclusive fashion in each of the LMS. The percentage of CRLM with highly expressed genes with TssA-promoters increased from 7% in LMS1 to 48% in LMS4, meaning that the fraction of open chromatin was markedly larger in the proliferative LMS4 compared with epithelial LMS1. In turn, closed chromatin associated genes with repressed and poised promoters had the biggest fractions in LMS1 and LMS5 with epithelial and endothelial characteristics, respectively. 

In summary, gene set analysis specifies the context of the LMS in terms of cellular programs related to epithelial- (LMS1), endothelial/mesenchymal- (LMS5), proliferative and virtually immune desert TME (LMS3 and 4), and immune cell composition, as well as chromatin states and CNV. These mechanisms markedly resemble molecular mechanisms observed in CRC-subtypes, suggesting that CRLM partly preserves the characteristics of the primary tumors. Pathway activities can be browsed interactively using the oposSOM web tool (see above). As worked example, activity patterns of the *WNT*-pathway for each LMS is provided in Supplementary Figure S7 showing specific activation in LMS2.

### 3.4. Trajectory Inference Indicates Continuous Alterations of Cellular States along Developmental Paths and Gradual Changes of the TME

**Figure 4:**
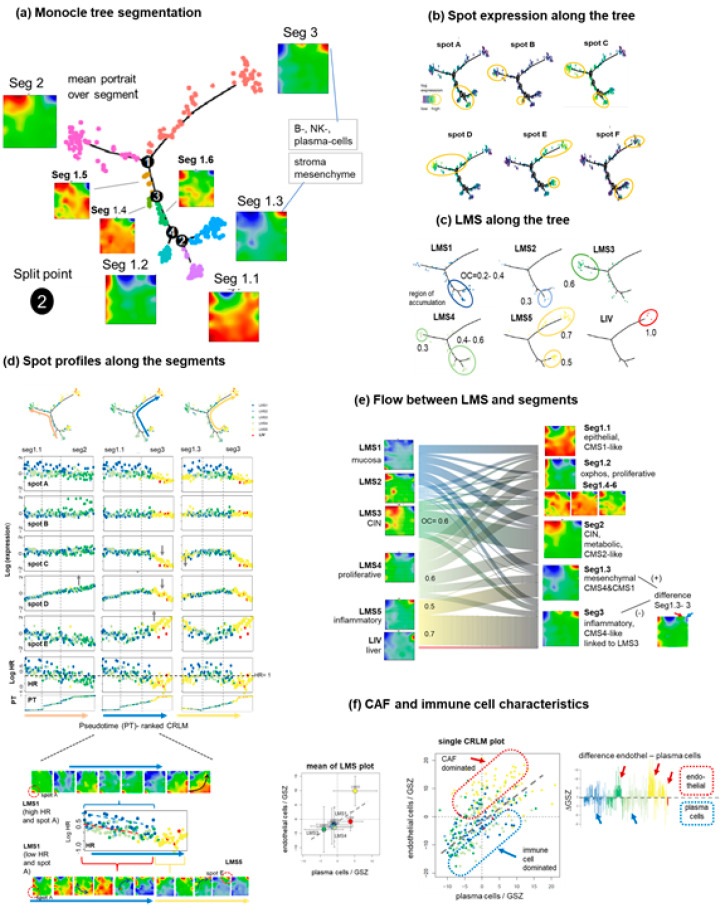
Tree trajectory analysis of CRLM heterogeneity using monocle [44]. (**a**) The monocle-tree divides into two major segments (Seg1–3) where Seg1 further splits into six subsegments (1.1–6). Mean expression portraits averaged over the CRLM portraits along the segments reveal changing expression patterns. (**b**) Samples along the tree are colored by spot expression A–F. Ellipses indicate areas of high expression of the respective spot. (**c**) Different LMS accumulate in different segments as marked by the ellipses and by the overlap coefficients (OC = overlap (LMS, segment)/(min_size (LMS, segment) between LMS and segments. (**d**) Profiles along different paths reveal smooth changes in spot expression. The log expression profiles of spots and hazard ratios (HR) use sorted CRLM along the abscissa. Pseudotime (PT)-scaled plots “virtually” compress the data along the subsegments 1.1 and 1.3 (Supplementary Figure S8a). Series of individual CRLM portraits from LMS1 and LMS5 indicate their pseudo-dynamics towards spot E (partly below and see also arrows in the portraits; for portraits of other subtypes see Supplementary Figure S8b). The blue and red dashed lines along the HR scatterplot visualize LMS1 HR-high and -low subgroups. (**e**) Sankey river-flow diagram between subtype and segment stratification of CRLMs. OC values greater than 0.5 show that LMS3 and, partly, LMS4 flow to Seg2, and LMS5 to Seg3 and Seg1.3. Their difference portraits resemble the epithelial patterns of Seg1.1 to overlay with Seg3 patterns. For interpretation of functional characteristics see Supplementary Figure S5 and next subsection. (**f**) Comparison of plasma and endothelial cell signatures (Figure 3e) in terms of biplots and their difference profile indicate that both signatures correlate but combine with different amplitudes in the CRLM (red and blue arrows and frames).

We performed trajectory analysis on the SOM portraits using the Monocle method [44], which sorts CRLM in low-dimensional space, an approach usually applied for inferring developmental paths in single-cell RNAseq settings. The application of this method to bulk expression data enables the extraction of potential paths of cancer progression [13]. The obtained tree divides into three major segments (Seg1–3) where Seg1 further subdivides into six subsegments (Seg1.1–1.6, Figure 4a). The mean portraits of the (sub)segments reveal systematic alterations of the expression patterns. To better characterize these changes, we visualize spot expression as well as the accumulation of the different LMS along the tree (Figure 4b,c, respectively). LMS2, LMS5 (partly), and LMS3 samples accumulate near the ends of Seg1, Seg2, and Seg3, respectively, paralleled by the high expression of the respective marker spots A, D, and E, respectively. LMS4 CRLM distributes over wider ranges of the tree and the tumors of LMS5 also accumulate in Seg1.3, which is also described with the expression of spot E.

To better understand expression changes along the three paths connecting different tip-points we generated spot profiles along the segments (Figure 4d). They reveal that the tree sorts the CRLM according to gradually changing expression levels, e.g., of the linearly increasing spot D expression from Seg1.1 to Seg2 opposed by its decay along Seg3, particularly associated with the accumulation of LMS5 (Figure 4d, middle column, Movie S1, a movie of the CRLM-portraits along the trajectory from Seg1.1 to Seg3 is provided in the supplementary file). Hence, tree analysis shows that the activity levels of biological characteristics such as epithelium (spot A), proliferation (spot C), CIN (spot D), and endothelium/inflammation (spot E) changes continuously and not abruptly between the CRLM, which possibly reflects developmental dynamics rather than static states. Interestingly, these continuous changes are overlaid by the typical differences between the subtype expression, e.g., of the high expression of spot A in LMS1 and of the lower expression in LMS2-4 along Seg1 and Seg2. 

Note that these expression differences associate with the overall survival hazard ratio (HR) and thus with prognosis, which also changes smoothly along the trajectories. Interestingly, CRLM samples of LMS1 split into two subgroups described with a high and low spot A expression and HR values. A series of individual portraits along the trajectory Seg1.1 towards Seg3 reveals that the expression patterns dynamically shift towards spot E, via the lower right part of the map (plasticity plateau, see next subsection), or rather, via its upper edge (Figure 4d, part below). Hence, the trajectories refer to (pseudo-) dynamically changing expression patterns beyond the spots, which overall illustrates the diversity of the transition states linking the states at the tips of the tree, which can be interpreted as archetypic expression states (Supplementary Figure S8).

The river plot visualizes the distribution of LMS between the segments (Figure 4e). A comparison of the mean portraits on both sides of the plots indicates more diverse and partly different spot patterns, particularly along Seg1, which suggests a fine granular activation of cellular programs in the CRLM not resolved in the LMS strata. One finds CRLM dominated by LMS1 in Seg1.1 and that LMS4 distributes broadly over Seg1.1, 1.2, 1.4–1.6, and 1.3, all of which show footprints of oxidative phosphorylation (oxphos) and proliferation. To better understand the bimodal split of LMS5 between Seg3 and Seg1.3 we calculated the difference portrait between them. It reveals that Seg1.3 can be understood as a superposition of the transcriptional programs of Seg3 and Seg1.2, i.e., of endothelial, epithelial, and proliferative gene functions, i.e., as a mixture of LMS3 and, to a lesser degree, of LMS1, which reflects a transition state between both LMS.

Moreover, Seg1.3 and Seg3 exhibit minor differences in the position of spot E in the right upper corner of the map, which also becomes evident in the difference map (see red and blue arrow in Figure 4e). Spot E is associated with the TME, which contains endothelial/CAF and inflammatory cells. The biplot of both compounds (where we selected plasma cells (PC) as a proxy of the immune compound) shows an overall linear relation between them with maximal values in LMS5 (Figure 4f, left part). The individual CRLM values considerably scatter with large deviations into positive (CAF dominance, red dashed frame and arrow) and negative (PC dominance, blue dashed frame and arrow) directions. These variations reflect relative PC-dominance in LMS1, partly in LMS4, and CAF dominance in LMS5 and partly in LMS3 (difference plot in Figure 4f, right part). 

When considering the marker genes of the other immune cells, we observe that B cells behave similarly to PC while mast cells more resemble CAFs, which overall reflects a CRLM-specific change in the cell communities of the TME (Supplementary Figure S9). Notably, the LMS5 tumors in Seg1.3 and Seg3 refer to a more endothelium/CAF-enriched and -depleted TME, respectively (Supplementary Figure S9). 

In conclusion, we observe a modified ordering of CRLMS along the monocle-tree, revealing continuously changing expression levels between the LMS, rather than clear-cut expression differences in most cases. This suggests developmental relationships among the tumors. Their ordering is governed by epithelial LMS1, CIN-affected LMS3, and immune cell-enriched LMS5 accumulating near the ends of the three major branches of the tree. These can be interpreted as archetypic expression states. LMS5 further splits into substrata enriched more with immune cells or CAFs, where the latter subgroup shares endothelial functions with LMS1 and also proliferative activity with LMS4.

### 3.5. High-Resolution Expression Cartography Deciphers an Interplay between Genetic and Epigenetic Regulation of Metastasis

**Figure 5:**
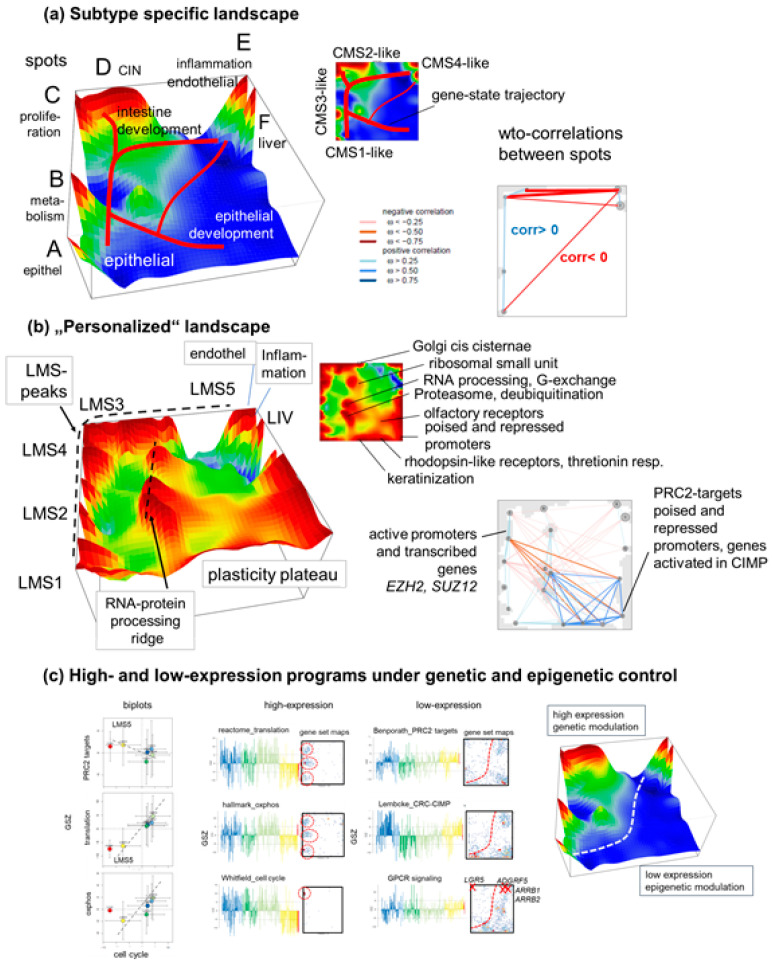
Transcriptomic landscape of CRLM. (**a**) LMS landscape expresses peaks A–F upregulated in a subtype-specific fashion. Blue regions assign areas of, on average, downregulated genomic programs in each of the subtypes. CMS-resemblance is shown in the small map. The weighted-topology overlap (WTO) network shows correlations between spots revealing that Spot E overall anticorrelates with the other spots. (**b**). The ”personalized” landscape considers upregulated spots of individual samples, independent of LMS subtyping. It revealed a more granular expression landscape and, particularly, a “plasticity plateau” related to various epigenetic mechanisms possibly promoting cellular reprogramming. The WTO network shows association between the programs, mostly demonstrating anticorrelation of the plasticity plateau with the proliferative programs near spot C. (**c**) Biplots of cell cycle expression signature [67] with *PRC2* targets [68] indicate overall negative correlations and positive correlations with translation and oxidative phosphorylation (oxphos) [36]. The genes of the latter functionalities accumulate in regions along the left edge of the map (red circles) while *PRC2* targets occupy specific areas in the right part, which, notably, agree with areas occupied by methylated genes of the CRC-CIMP type [69] and also G-protein-coupled receptors (Reactome GPCR-signaling).

Next, we sought to better understand the fine-grained details of the expression landscape that became visible along the monocle tree. The LMS-averaged mean portraits provided a transcriptomic landscape with six major overexpression spot modules A–F, each associating with a specific functional context and a specific differential expression in virtually all of the subtypes (Figure 5a and also Figure 1 and Figure 2). The oposSOM software offers an alternative segmentation of the expression landscape, such as through a personalized summary map. This approach leverages the individual expression portraits of CRLM, thus enabling the detection of less frequent and subtler overexpression patterns independent of LMS classification (see Supplementary Figure S10 for details). This method reveals a richer, more structured overexpression landscape with additional details. These include a “plasticity plateau” in the right lower corner as well as an “RNA-protein molecular processing ridge” of modules assigned to functions such as RNA-processing, de-ubiquitination, and proteasome in the middle of the map (Figure 5b). The former plateau is associated with the epigenetic mechanisms of cellular reprogramming into plastic states, such as poised and repressed gene promoters in colonic tissue, targets of *PRC2* (polycomb repressive complex 2) and of the CpG island methylation phenotype (CIMP) [45,46,47], the genes responsive for thretionin treatment via rhodopsin receptors [48], and gene signatures related to keratinization [49,50] (Supplementary Figure S5). The “processing ridge” shows distinct expression modules associating with CRC- and CRLM-related mechanisms, such as protein degradation and de-ubiquitination [51,52,53], RNA-processing, guanyl-exchange [54], and ribosomal assembly [55], as well as the adaptation of the Golgi apparatus in cancer [56].

Weighted-topology overlap (wto-) networks [57] between the spots reveal anticorrelation between spot E and the other spots on the one hand and between the plasticity plateau and the proliferative spot C on the other, i.e., overall between the left and right side of the map (see the wto-correlation maps in Figure 5a,b). This reflects the antagonism between the inflammatory and endothelial contexts (spot E and LMS5) and the epithelial and proliferative functions (spot A–D). Indeed, biplots of the gene set activities “cell-cycle” versus “*PRC2*-targets” reflect a negative slope between LMS5 and the other LMS while the correlation of “cell cycle” versus “translation” and “oxphos” is positive (Figure 5c, left part). Profiles and maps of these gene sets provide further interesting details (Figure 5c, right part): genes of the set “cell cycle” accumulate in spot C and genes of the sets translation and oxphos accumulate in the three characteristic areas, including spot B (see also Supplementary Figure S5). All these functions associate with low expression in LMS5 and high expression in the other LMS, however with subtle mutual differences, e.g., the moderate downregulation of oxphos activity in LMS3. 

The genes *EZH2* and *SUZ12*, both encoding components of the *PRC2* complex, also locate near spot C, while, in contrast, the *PRC2*-target genes accumulate in characteristic areas in the right part of the map near spots E, partly A, and in the “plasticity plateau”. They are associated with upregulation in LMS5 and partly in LMS1. Genes activated in the CIMP (CpG island methylator phenotype) accumulate in similar regions, thus suggesting the promoter hypomethylation of *PRC2*-targets. Similar gene accumulation patterns in the right part of the map were found for repressed and poised promoters in the colon as well as so-called low-expression transcription factors (low-TF) which associate with virtually deactivated chromatin states in a wider sense (Figure 5c and Figure S5e) [58]. It was recently reported that the downregulation of *EZH2*, the catalytic subunit of *PRC2*, results in increased DNA replication initiation through the loss of repressive histone methylation marks from bivalent, poised promoters retaining the ability to be activated as differentiation proceeds [59]. It involves thousands of genomic loci of DNA replication origins scattered throughout the genome that associate with open euchromatin regions and the regulation of pluripotency and differentiation-related genes. In contrast, so-called high expression-TF genes and active promoter states accumulate in the left part of the map including spots B–D, in activated cycling and metabolically active states. Importantly, key mutated genes in CRC and CRLM also locate in the left high-expression part of the map in support of their driver function for transcriptome programs (Figure 1c). The bimodal expression of repressed/poised versus active/transcribed chromatin states, evident in the heatmap in Figure 3k, thus transforms into virtually left-to-right antagonisms of gene expression in the SOM landscape. 

Comparing the averaged expression portraits along the tree segments (Figure 4a) with the personalized landscape (Figure 5b) enables the inference of a trajectory in the gene-expression landscape [60]. Accordingly, it links expression modules related to epithelial functions (Seg1) with intestine-developmental (Seg2) and inflammatory and mesenchyme (Seg3) functions. Moreover, it suggests an additional path through the plasticity plateau towards the LMS5 tumors accumulating in Seg1.3 (Figure 5a). Interestingly, this “epigenetic” path includes a region enriched in G-protein-coupled receptors (Figure 5c), which play a crucial role as signaling transducers in the development of CRC [61] in promoting EMT [62], where particular G-protein-coupled receptors (GPCRs) such as *ADGRF5* [63] and *ARRB1* and *ARRB2* [64] were identified as EMT- and poor survival markers in CRC. These genes locate near spot E and upregulate in LMS5. The GPCR *LGR5* as an exception locates in the high expression side of the landscapes in spot C, upregulated in highly cycling CRLM, which agrees with the role of *LGR5* as a marker for proliferative stem-like cells in CRC [65,66]. In summary, high-resolution cartography of the CRLM transcriptomes reveals a network of cellular programs governed by genetic lesions (mutations and CNVs) and by epigenetic chromatin remodeling leading to the antagonistic activation of epithelial-like proliferative cancer cells or inflammatory, mesenchyme-like cells. This provides rationales for the sequential activation of transcriptional programs and the ordering of CRLM along the segments of the monocle-tree.

### 3.6. Portrayal of Clinical, Mutational, and Telomere Maintenance Characteristics 

**Figure 6:**
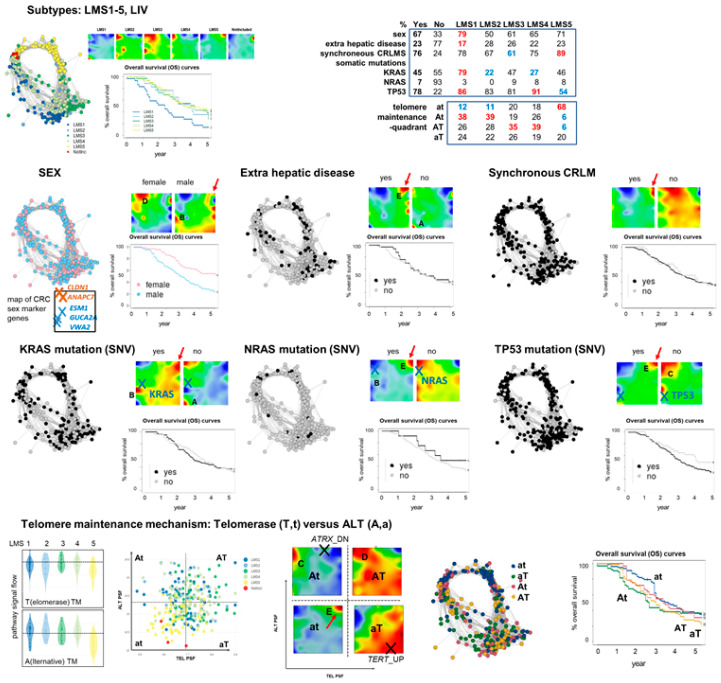
Subtypes, selected clinical characteristics, somatic mutations, and telomere maintenance pathway activation. The respective cases were shown in sample space as similarity nets, as mean portraits averaged over the respective samples, and in terms of prognosis using overall survival (OS) curves. The table lists the percentages of CRLM in the respective strata (data were taken from [5]). Gene locations are mapped as crosses in the portraits. Sexual marker genes were taken from [75]. The red arrows indicate upregulated spot E (inflammation). Analysis of telomere-maintenance mechanisms includes violin plots of the pathway signal flow (PSF) activity of telomerase (T) and alternative (A) telomere maintenance using TM pathways from [76], a biplot of T- and A-TM PSF values and their stratification according to the four quadrants (a, t…low values, A, T…high values), similarity plot, mean portraits, and OS-curves.

Next, we characterized the CRLM after stratification into the different LMS with respect to selected clinical items and mutations, as well as telomere maintenance (TM) pathway activities by means of SOM portrayal and overall survival (OS) curves (Figure 6). LMS1 has an inferior prognosis compared with the other LMS2-5 with a virtually identical prognosis (see also [5]). These OS-relations partially align with the comparable CMS, namely the inferior prognosis of CMS1 CRC compared with CMS2-4 [70]. However, this deviates from other studies that reported a poorer prognosis for LMS5-resembling CMS4 [24,28], possibly due to the early stage, non-metastatic nature of these CRC cohorts. Furthermore, males, compared with females, showed a higher incidence (67% versus 33%) as well as an inferior prognosis. This sexual bimorphism is in-line with previous reports on CRC [71,72,73] and liver metastases possibly due to the regulation of the TME [74]. The sex-stratified portraits indicate the increased expression of spot D (CIN) in women and of spot B (*PRC2*, metabolism) in men together with an increased expression level of the plasticity plateau. Sex-specific biomarkers for CRC taken from [75], located in spot D (*CLDN1*, *ANAPC7*), are upregulated for women, and in/near spot B (*ESM1*, *GUCA2A*, *VWA2*) are upregulated for men, which further supports the analogy between CRC and CRLM. 

Extrahepatic disease accompanying CRLM and synchronous metastasis (within 6 months of CRC diagnosis) are both associated with an average upregulation of spot E (inflammation). *KRAS* and *TP53* somatic mutations associate with the expression changes of spot B, which harbors both genes in its neighborhood, acting in opposite directions, however, namely in up- and downregulation, respectively, which is in accordance with the major functions of *KRAS* as oncogene and of *TP53* as a tumor suppressor. These mutations, as well as that of *NRAS*, on average activate spot E and thus the endothelial immune responsive LMS5 and, on the other hand, associate with the downregulation of proliferation and CIN characteristics (spot C and D, respectively) and of the epithelial signature (spot A) for mutated *KRAS*. The mean LMS5/spot E characteristics of CRLM with mutated *KRAS* and/or *TP53* align with the increased mutation frequencies of these genes along a hypoxia axis that associates with the activation of spot E [3].

Finally, we compared the pathway signal flow (PSF) activation values of the telomere maintenance pathways via telomerase-dependent (TEL) and alternative (ALT) mechanisms (Figure 6 below, [76,77]). The ALT-versus-TEL activity biplot shows that the double-low quadrant “at” (a…ALT-low, t…TEL-low) enriches LMS5 with the marked upregulation of spot E and downregulated proliferation of spot C. The “At” quadrant (A…ALT-high, TEL-low) associates with activated proliferation (spot C), while the “aT”-quadrant upregulates the plasticity plateau. Poor and better prognoses associate with “at” and “At” quadrants, respectively, i.e., with the vertical ALT-axis. Interestingly, the high-ALT “At” quadrant and the high TEL quadrant “aT” associate with the downregulation of *ATRX* and the upregulation of *TERT*, respectively (see gene locations marked by crosses in the At and aT portraits), which are also known to switch-on ALT and TEL TM in other cancer types, such as gliomas [78]. In summary, the stratification of CRLM according to selected clinical items, the mutations of key genes as well as TM, reveals a dualism of expression between LMS5-like and non-LMS5-like patterns with activated spot E versus spot A and C, respectively, which reflects the overall antagonism between mesenchyme/inflammatory and proliferative/metabolic cellular functions where, e.g., mutated (*TP53*, *KRAS*) CRLM are more prone to the former states.

### 3.7. Prognostic Maps Provide an HR-Score along the EMT-Axis Related to Treatment

**Figure 7:**
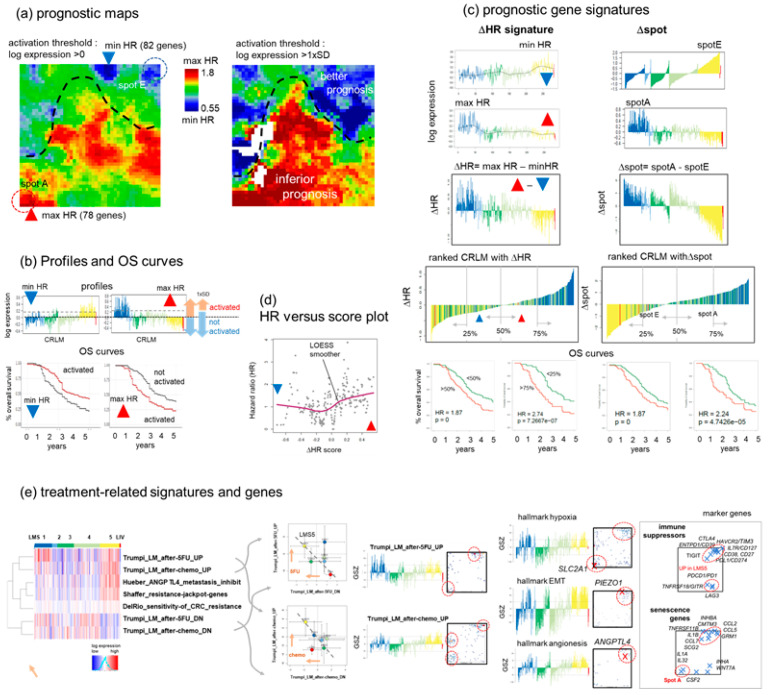
Prognostic maps and scores and their relation to treatment and the epithelial–mesenchymal transition (EMT). (**a**) The prognostic map color codes associations between metagene expression and the hazard ratio (HR) relative to the overall survival averaged over all CRLM in red (high expression, high HR) to blue (high expression, low HR). The left map refers to a lower and the right map to a higher expression threshold. Metagenes of maximum and minimum HR (HRmax and HRmin, respectively) are indicated by red and blue triangles in the left map. (**b**) Profiles of the HRmin and HRmax metagene expression. The overall survival (OS)-curves of patients with activated and non-activated expression are virtually mirror symmetrical for HRmin and HRmax regarding good and inferior prognoses. (**c**) Profiles of genes from HRmax and HRmin metagenes are combined into a ΔHR score. Positive and negative ΔHR values were strongly enriched within LMS1 and LMS5 CRLMs. A 25-percentile selection along the score provides slightly more discriminative OS curves than the 50-percentile selection into good and bad prognosis CRLM. Using genes of spots A and E for the score (Δspot) provides similar results. (**d**) The HR-versus-(ΔHR-)score plot reveals, on average, a non-linear relation where HR increases virtually only at larger score values at increased LMS1 content. (**e**) Gene set analysis supports the functional interpretation of neoadjuvant chemotherapy treatment resistance and bad prognosis in terms of EMT: the heatmap considers genes promoting metastasis via an angiogenic mechanism [88], “jackpot” genes of treatment resistance driven by epigenetics [89], genes upregulated after FOLFIRI treatment [90], and genes up- or downregulated after chemotherapy of CRLM patients and in vitro 5-Fluorouracil (5-FU) [6]. x–y biplots of the treatment effect (x—downregulated genes, y—upregulated genes) show a shift towards immunosuppressive LMS5. The up-profiles and gene maps resemble those of the hallmark hypoxia [36], promoting vicious and immuno-suppressive metastasis [3]. Hallmark genes of EMT- and angiogenesis accumulate in spot E together with immune checkpoint inhibitors [81] and senescence genes which facilitate resistance against immunotherapy [82].

We used the survival data from the cohort to generate prognostic maps, which display the hazard ratio (HR) for each pixel (metagene) in a red to blue color scale. The HR active status was estimated by applying two threshold settings for overexpression: either larger than the mean or one standard deviation away from the mean of metagene expression (Figure 7a,b and [11] for details). Inferior prognosis (indicated by red areas in the map) is associated with the upregulation of genes in and around spot A and the processing ridge in the middle of the map. These overexpression states are predominately found in LMS1. Good prognosis (blue areas) is found around spot E, associating with LMS5, and also spot B, C, and D upregulated in LMS2-4. A detailed comparison of the prognostic maps with the overexpression maps reveals that metagenes of maximum and minimum HR (HRmax and HRmin, respectively; for lists of genes see Supplementary Table S4) slightly deviate from the overexpression spot-areas of maximum expression, particularly of spot A and E, respectively. Each of those metagenes contain about 80 transcripts (red and blue triangles in Figure 7a). Their profiles upregulate in LMS1 (maxHR) and LMS5 (minHR), respectively (Figure 7b). We defined a prognostic score by calculating the log-expression difference (ΔHR = maxHR − minHR), ranked the CRLM with increasing ΔHR-score, and calculated the OS-survival curves for the high- and low-risk groups (top and bottom 50% or 25% of CRLM), which resulted in OS-curves resembling those between LMS1 and the other three subtypes (compare with Figure 6). 

Substituting the genes taken from the maxHR and minHR metagenes by genes of spot A and spot E results in similar OS-curves, indicating that limiting the prognosis of CRLM patients is well reflected by the subtypes LMS1 (most inferior) and LMS5 (better). Key genes frequently mutated and/or deregulated in CRC and CRLM, including *APC*, *FBXW7*, *SMAD4*, *PIK3CA*, *WNT5A*, and *ATRX*, are found adjacent to the minHR metagene, suggesting that their upregulation is associated with better prognosis (compare Figure 7a, left map and Figure 1c). Further, the minHR area enriches genes of the set “Golgi cis-cisternae” (gene ontology (GO) cellular component) playing a role in sustaining invasion and metastasis such as the intracellular signaling platform, lipid biosynthesis, protein secretion, the formation of extracellular vesicles, and the overall supporting of the adaptation of cancer cells to acquire a mesenchymal-like migratory phenotype via extracellular matrix remodeling ([56] and references cited therein). The maxHR regions are enriched in genes of the epithelial functional context and bad prognosis in cholangiocarcinoma [79] as well as nasopharyngeal- [80] carcinomas (Supplementary Figure S5). A plot along the ΔHR score (after averaging and smoothing) indicates that HR increases, on average, only at larger values of the score, which are enriched with LMS1 cases (Figure 7d).

The expression profiles of gene signatures related to neoadjuvant chemotherapy and treatment resistance in CRC and CRLM show activation in LMS5 and partly in LMS1 suggesting a treatment- and metastasis-driven shift of function towards an immuno-suppressive phenotype (Figure 7e, left part) [6]. A detailed inspection of the signature profiles and their gene maps reveals close similarity with those of the hallmark set “hypoxia” which was recently identified as a driving process in CRC liver metastasis and chemotherapy-treatment resistance (Figure 7e, right part) [3]. The hypoxia signature genes distribute over spots E and A, where the latter hosts the key regulator *SLC2A*, which promotes immunosuppression via M2-macrophage enrichment combined with immune escape via specific CD4+ T-cell exhaustion [3] (Figure 7e, right part). The virtually identical profiles and maps of the hallmark genes “epithelial-mesenchymal transition” (EMT) and “angiogenesis” reflect the close relations between these functions: both blood vessel formation (angiogenesis) [81] as well as senescence in a secretory cell phenotype [82] facilitate metastasis and treatment resistance. The respective marker genes accumulate in and around spot E together with immuno-suppressors and hallmark “EMT” genes thus indicating co-regulation. Interestingly, the *PIEZO1* gene, coding a mechano-sensitive sensor membrane-channel protein also locates in the endothelial/CAF-rich left region of spot E. The mechano-sensitivity of tumor and of TME-cells such as macrophages is important for their ability to transduce mechanical forces into biochemical signals associated with signaling pathways involved in cancer metastasis and EMT, such as angiogenesis, cell migration, intravasation, and proliferation [83,84,85], as well as cytokinesis and endosome trafficking [86]. The proper processing of mechanical stress is an essential function to master the changing cell architecture and extracellular-matrix (ECM) organization along the EMT-path [87].

In conclusion, the prognostic maps divide the expression landscape into regions of better and worse prognosis, which are associated with the functional context of the affected spots assigned above. These maps enable the extraction of a prognostic expression score along the EMT-axis between spots A and E and their adjacent regions. Treatment resistance, immune evasion, and a series of EMT-related processes such as hypoxia, angiogenesis, senescence, and micro-mechanic sensitivity associate with neoadjuvant treatment and the formation of an immuno-suppressive, mesenchymal state of CRLMS.

### 3.8. Intra-Patient Heterogeneity of Metastasis

**Figure 8:**
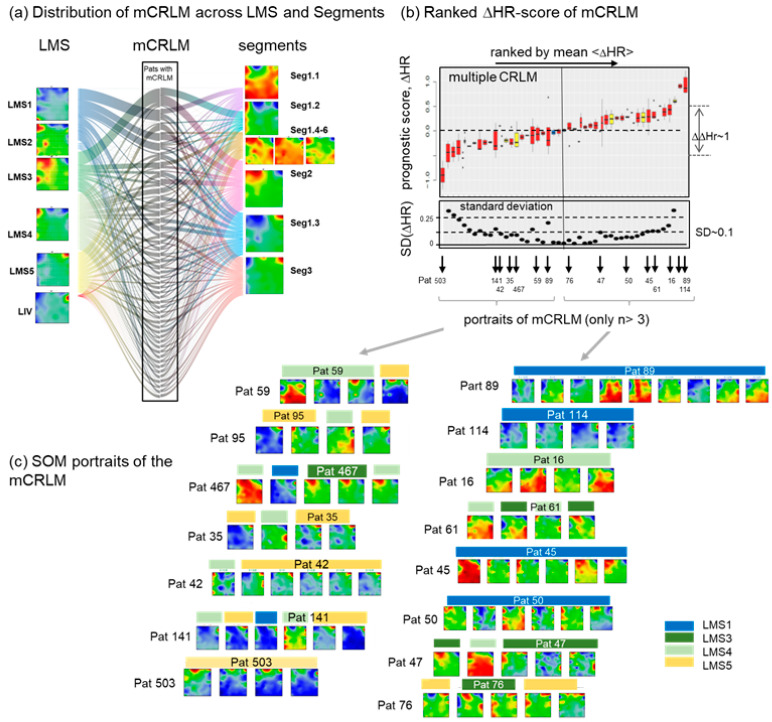
Intra-patient heterogeneity of CRLM: (**a**) Flow diagram of multiple CRLM (mCRLM) from the same patient towards LMS and segment classifications to the left and to the right, respectively. (**b**) Mean ΔHR score of the mCRLM averaged per patient and ranked from the left to the right. The boxes refer to the standard deviation (SD) around the median per CRLM and enlarged plot of the standard deviation. (**c**) SOM portraits of the mCRLM per patient sorted by part (**b**) (see patient no. and arrows). The color bars assign the LMS.

More than one CRLM samples were collected from nearly fifty patients at different time-points, [5] providing a set of multiple CRLM (mCRLM) which enables a glimpse of the intra-patient mCRLM heterogeneity. A river flow plot links the intra-patient mCRLM with their LMS and segment classes revealing no unique assignment in many cases (Figure 8a). For an alternative perspective, we applied the continuous ΔHR prognostic score for heterogeneity analysis in terms of a mean-value ranked plot of the mCRLM (Figure 8b). Its slope and vertical spread closely resemble the respective plot of the individual CRLM (compare with Figure 7c). The standard deviation of ΔHR for each mCRLM overall forms a relatively narrow range of intra-patient variability (see box-heights or separate plot in Figure 8b below). 

The overall inter-patient heterogeneity of the prognostic score exceeds the intra-patient heterogeneity by roughly one order of magnitude, considering the change in ΔHR across its linear range of the plot (ΔΔHR ~1) as a measure of the former and the mean SD (~0.1) as a rough estimate of the latter effect. About 50% of the mCRLM from the same patient belong to different subtypes [5], a result that suggests a relatively large intra-patient heterogeneity in contrast to our estimation. Inspection of the individual portraits (with more than three mCRLM per patient) reveals that part of the mCRLM of the same patient with different LMS memberships show very similar expression patterns (see, e.g., pat. 467, 141, 61, 47, Figure 8b). Moreover, the distinction between LMS2–LMS3 is relatively uncertain, which then also applies to the heterogeneity estimation based on LMS-membership (Figure 1d and Figure 2c). 

On the other hand, the SD increases towards the end of the ΔHR scale. Selected portraits from these regions (e.g., pat. 503 from the left and pat. 89, 16 from the right side) show relatively diverse patterns despite their unique LMS assignment, suggesting a relatively high variability of the underlying transcriptional states, particularly in the low-expression epigenetic effects. Notably, the portraits of the right, high inferior prognosis side reveal an enrichment of CRLM with activated “plasticity plateau” referring to high (red) ΔHR areas in the prognostic map (Figure 7a). In summary, the transcriptomes of mCRLM from the same patient are relatively homogeneous based on the prognostic ΔHR score and its variability. mCRLM of inferior prognosis is variant regarding their epigenetic context showing either the activation of spot A (epithelium) and/or of the plasticity plateau.

## 4. Discussion

### 4.1. Subtyping Stratifies CRLM along Cancer Hallmarks and TME Education

**Figure 9:**
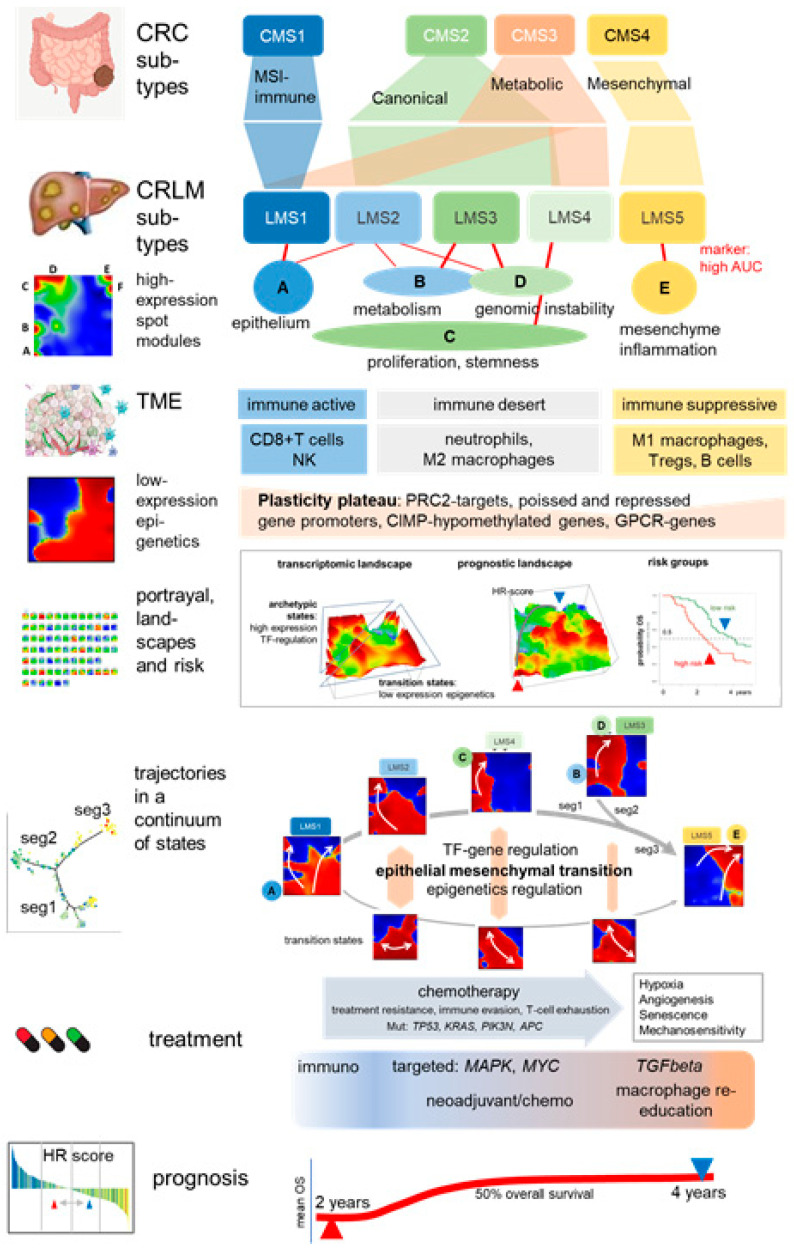
A holistic view on colorectal cancer liver metastasis: molecular heterogeneity, tumor microenvironment, genetic and epigenetic modes of regulation, prognosis, tumor development, and possible treatment options. See discussion.

We reanalyzed transcriptome data of liver metastases using SOM portrayal, a machine learning method that generates individual images of each tumor sample, reduces the dimensions of the transcriptome-wide expression data, and enables straightforward and intuitive downstream analysis [21]. The previously identified five LMS [5] were used to provide subtype-specific SOM portraits revealing six major modules of co-regulated genes. These related to molecular hallmarks of cancer, namely avoiding immune destruction (spot A), tumor-promoting inflammation and angiogenesis (spot E), sustaining proliferation (spot C), deregulating cellular energetics, metabolic activity and DNA repair (spot B), and CIN-related genomic instability (spot D) (Figure 9) [91]. The spot-genes provide robust signatures for classifying the LMS, meeting a trade-off criterion of balancing the interpretability and specificity of molecular markers [92]. 

The functional characteristics of the LMS align with known molecular CRC subtypes, suggesting that metastasis, at least partly, maintains characteristics of primary tumors, which could inform subtype-targeted therapy for both metastatic and non-metastatic tumors. A high concordance of genetic key lesions, mutations, and CNV between primary and metastatic tumors supports this observation. This suggests that there is minimal impact of the liver microenvironment on CRLM mutation patterns, but rather neutral evolution mechanisms of early metastasis due to the high fitness of the metastatic clones [3,27]. On the other hand, the liver facilitates metastatic expansion from other organs because of its complex immune system that dampens immunity to cancer-related neoantigens, promotes immune editing, and changes TME-characteristics of the metastatic compartment compared with the primary tumors [7,93,94] thus offering a tumor-supporting (pre-)metastatic niche [95]. Interestingly, a recent PanCancer metastasis study on 4000 metastatic tumors across thirty-two cancer types taken from the cancer genome atlas (TCGA) reported four major molecular groups of metastasis (s1-4) [10] partly associating with the function of our spot modules, namely proliferation, DNA repair and CIN (spot C and D) in s1, metabolism (spot B and C) in s2, and inflammation and immunity (spot E) in s4. The epigenetic subtype s3 can be related to the ”plasticity plateau” and, partly, spot B-accumulating *PRC2* targets observed across all LMS with elevated expression. Extensive drug testing in cell lines has suggested therapeutic options for s1 (e.g., targeting *MYC*) and s3 (e.g., targeting histone acetylation and methylation as well as *TERT* and *EZH2*). Potential treatment options for primary CRC were recently reviewed [96] and include immune and adjuvant therapy depending on the CIMP-status for CMS1 (and possibly LMS1), targeted treatment to *MAPK*- and *WNT*-pathways, *MYC*-expression or glycolysis metabolism for CMS2 and/or CMS3 (and possibly LMS2-4), *TGFbeta*, and also cancer stemness inhibitors for CMS4 (and possibly LMS5). We also studied telomere maintenance pathways in CRLM and found decreased activity in both TEL and ALT TM in LMS5, but increased ALT, which directly correlates with the upregulation of spot C (proliferation, stemness). Changes in TM activity are a hallmark of cancer modulation in CRC [77] and are suggested as markers of response to therapy [97].

Our analysis identified factors driving CRLM characteristics towards the immune-suppressive spot-module E, namely accompanying extrahepatic disease(s), synchronous metastasis, and *TP53*, *NRAS*, and *KRAS* mutations, where the frequency of these mutations is reported to increase in immuno-suppressive immunotypes expressing spot E in our data [3]. Moreover, neoadjuvant chemotherapy also induces a shift of CRLM and of derived tumor cell cultures towards a mesenchymal phenotype [5,6], which is supported by the LMS5-resemblance of the extracted expression signature. However, it is important to note that cancer therapies, particularly platinum-based/5-FU therapies, can cause significant changes in the tumor genome, and introduce an evolutionary bottleneck that selects for known therapy-resistant drivers [98] and induces mutational burdens exceeding aging-related mutations accumulating in the same time-span by several orders of magnitude [99]. However, the impact of this mutational “toxicity” on tumor development is not entirely clear. Early CRC drivers were found to be enriched in CRLM, which can harbor private mutations (*PTPRT*, and to a lesser degree, *AMER1* and *TCLF1*), also suggesting stringent evolutionary selection mechanisms for the part of the CRLM [27] that is possibly governed by treatment [25]. 

Genetic alterations and chromosomal instability are necessary but insufficient for cancer initiation, progression, and metastasis. Instead, tumors, and particularly CRLM, are rather complex ecosystems involving cancer cells and the TME [94]. Our analysis suggests an interaction between the TME and stroma-mediated immune suppression and treatment resistance during CRLM development. Using a series of TME-related signatures, as well as the single-cell deconvolution of bulk expression data, we found indications of an immunogenic pro-tumoral and tumor-suppressive TME in LMS1 and LMS5, respectively, and of predominantly immune desert properties in LMS2-4. However, part of the CRLM of the immune-desert subtypes show a medium-level expression of spot E, thus also reflecting moderate inflammatory, immuno-suppressive characteristics in these LMS. We found that spot E overlays signatures of cancer-associated fibroblasts, of the stroma and of different immune cells such as B-, plasma, pDC, and mast cells reflecting the overall considerable heterogeneity and changing composition of the TME-cell communities not explicitly resolved in the LMS stratification. This suggests the education of the TME towards stroma-mediated immune suppression and treatment resistance upon CRLM development [100,101]. Further, spot E co-expresses with a series of immune checkpoint inhibitors such as *CTLA4*, *PDL1*, and *TIM3*, whose increased expression associates with T-cell exhaustion, macrophage re-education from M2 towards M1, and decaying CD8+ expression in agreement with [3,7,102]. The central role of tumor-associated macrophages (TAMs) in the TME is substantiated by the evolutionary consequences TMA impose through positive or negative selective pressure exerted by/on tumor cells [35]. TAM complexity is paving a possibly way for the design of novel TAM-targeting therapies to harness their anti-tumoral potential [103], e.g., through *CCR5*-inhibitor treatment reprogramming TAMs toward a pro-inflammatory phenotype in metastatic CRC patients [104]. Overall, transcriptomic portrayal confirms the previous subtyping of CRLM, deepens the understanding of their functional context, provides marker signatures for the LMS, and delineates the remodeling of the TME from an immuno-active to an immuno-suppressive state. 

### 4.2. Trajectory Inference and Personalized Analysis Discover a Hidden Universe of Continuous Epigenetic States Shaping Metastasis

LMS 1-5 subtypes were identified by class discovery using non-negative matrix factorization followed by cluster number optimization based on genes with high specific expression levels across the subtypes [5]. Consequently, the LMS-specific spot markers were biased towards highly variant genes, which can unintentionally neglect lesser variant gene clusters. Our personalized landscape-approach considers individual portraits beyond the LMS-strata and therefore extracts additional features appearing as weakly variant spot modules not detected in the LMS-centered analysis, akin to a “dark matter” of gene expression (in analogy to the not-visible matter in the universe). 

Notably, our approach reveals the activation of regulatory programs with epigenetic impact such as *PRC2*-targetted genes with poised and repressed promoters in a healthy colon which change their promoter status in CRLM through chromatin remodeling [17]. We found that these epigenetic modes antagonistically regulate compared with a part of the high-expression programs including, e.g., genes with active promoters governing functions such as cell cycle, DNA repair, and oxphos metabolism. The activation of such epigenetic modes is associated with the ability of cancer cells to acquire plasticity regarding their fates. It enables them to transit between high-expression states and low-expression states, such as along the epithelial–mesenchymal transition axis taking place between LMS1 and LMS5. We observed such epigenetic states in all subtypes, which suggests that all of them are prone to fate decisions via plasticity-driven changes in their state. 

The balancing between active and proliferative genes on the left, and repressed, plastic chromatin states on the right enables evolutionary adaptation to the TME and drives not only the adenoma-to-carcinoma transition of CRC but also promotes metastasis, e.g., via the EMT [45]. Components of the *PRC2* such as *EZH2* and *SUZ12* (located in spot C) can facilitate these transitions and promote CRC stem-like cells (as indicated by the stemness marker *LGR5* in spot C, see below) via repressive interactions with their targets (located in the plasticity plateau) in concert with changes to histone and DNA methylation in CIMP genes. Notably, the bimodality between active and repressed chromatin states and their association with proliferative and immunogenic states was described for other tumors such as lymphomas [92] and gliomas [11]. In a more general sense, the interplay between high-expression-genetic and low-expression-epigenetic states, as identified in the CRLM expression landscape, is a key condition for the reorganization of transcriptional programs that facilitate tumor development via the emergence of new attractor states better adapted to the changing TME [8]. Epigenetic mechanisms also affect antitumor and protumor immunity and the TME immune and CAF cell composition, thus facilitating immune state transitions during tumor development [103]. An interesting argument supporting the role of epigenetics in metastasis comes from the result that mutational patterns of passenger mutations in metastatic tumors outperform those of driver mutations in predicting their primary tumor of origin, presumably because the regional density of somatic passenger mutations reflects chromatin accessibility to DNA-repair complexes, which in turn relates to the epigenetic state of the cancer cell [105]. 

Trajectory inference revealed possible developmental paths from epithelial (as seen in LMS1) to mesenchymal (in LMS5) characteristics, which could lead to enhanced metastatic fitness through an interplay of genetic and epigenetic factors [106]. These trajectories are governed by continuous alterations in spot expressions suggesting that the LMS subtypes are “connected” through a continuum of intermediate states. This continuum combines the “archetypic” LMS characteristics and epigenetic regulatory modes such as the plasticity plateau with the LMS spots in diverse ways and with varying degrees of influence. Further, the transition to metastasis is thought to be facilitated by the presence of proliferative CRC cancer stem cells (CSCs). These CSCs constitute presumably a small population of highly tumorigenic cells, possessing pluripotency and self-renewal properties that drive metastasis and treatment resistance. This process is sustained by a dynamic TME [107]. Complex interactions between the TME and CSC not only maintain stemness but also fuels tumor evolution into aggressive, invasive, migratory phenotypes [100], a process thought to be under the influence of epigenetic control [108]. The CSC marker, *LGR5*, also a marker for normal intestinal stem cells, suggests that the stem-cell program in the colon could be conserved in CRC [106,109] and CRLM. Additional factors such as CRC-typical CIN (e.g., in spot D), which boost metastasis, amplify oncogenic signaling, leading to more aggressive cancer clones, punctuated tumor evolution [110], and immunoediting in the TME context [111].

The oscillation between hallmark-related archetypic genetic and plastic epigenetic states suggests that high-transcription activity stabilizes the genetic states, while low-transcription promotes transitions in response to developmental or environmental cues [112]. Open chromatin states, associated with high expression, may inhibit differentiation and promote tumor growth via proliferative, metabolic, and CIN hallmarks, primarily in LMS2-4. Conversely, permissive or “plastic” states may allow cell fate transitions, providing a fitness advantage along the EMT-trajectory from Seg1.1 (mainly LMS1) to Seg3 (mainly LMS5) of the monocle tree. We recently showed in melanoma cell lines that targeted treatment can shift cell physiology from high-transcription to plastic, low-transcription epigenetic states [15]. In summary, combining trajectory inference with Self-Organizing Map (SOM) portrayal reveals a continuum of transcriptomic states that divides into archetypic hallmark-states and intermediate transition states driven by epigenetic plasticity and characterized by diverse combinations of these characteristics. 

### 4.3. Towards Precision Diagnostics and Treatment Decisions of CRLM

This dual continuous and plastic characteristic of CRLM heterogeneity necessitates a fine granular diagnostic scheme that surpasses the somewhat generalized LMS stratification. Our portrayal method provides unique molecular images of each tumor, enabling personalized diagnosis through the intuitive visual interpretation of spot patterns, which are interpretable in terms of activated cellular programs. Hence, these personalized portraits can be evaluated by experts in a diagnostic pipeline to assess the specific transcriptional state of a CRLM sample. Such “manual” inspection of molecular portraits could be automated, for example, through neuronal network machine learning, in future stages of technical development (see [92] for a proof of principle application to another cancer type). Moreover, transcriptomic maps can be transformed into prognostic landscapes visualizing the association between gene expression, cellular programs, and their corresponding HR. To adequately account for the continuous nature of CRLM heterogeneity within the transcriptomic landscape, we applied a one-dimensional HR-score along the EMT axis as a proof of principle solution that links the best and worst HR metagenes in the landscape. This scoring method can be extended to multidimensional molecular coordinates, as identified by the monocle-tree analysis or by combining different spots for prognostics within a coordinate system of continuous scores [113].

Another implication of the continuous developmental trajectories of CRLM, which are governed by epigenetics, is that treatment will require molecular surveillance and adaptation to evolving states. The dynamic cellular composition and functional characteristics of the immune landscape along the trajectory of cancer development are expected to impact therapeutic efficacy and clinical outcomes. Targeting epigenetic modifiers to remodel the tumor-immune microenvironment holds great potential as an integral part of anticancer regimens [103]. 

The so-called oligometastatic hypothesis provides an alternative perspective on the development and treatment of metastasis, suggesting that the early and aggressive treatment of metastases may prevent the further spread of cancer and even achieve long-term remission [104]. When applied to CRLM, it shows that a canonical, proliferative subtype resembling LMS3-4, with an elevated CIN-signature score [114] of genes accumulating in and around spot C is associated with increased metastatic propensity, and inferior overall survival [115,116,117] (Supplementary Figure S5d). Surprisingly, a specific subgroup of these CRLM, also with a high CIN-score, exhibits the opposite effect. They demonstrate a therapeutic vulnerability to DNA-damaging therapies, leading to improved treatment responses and low clinical risk with a 10-year survival rate of 95% after resection [116]. However, the challenge remains how to differentiate high-CIN-score tumors likely to respond to DNA-damaging therapies from those unlikely to respond. Our results show that CRLM with an upregulated CIN-score (spot C, proliferation up) widely distributed over LMS1–LMS4 with a broad spectrum of functional and genomic characteristics associated with activated proliferation. This provides a basis for further studies to distinguish responders from non-responders to DNA-damaging therapy.

### 4.4. Limitations and Open Points

Our analysis has several key limitations. Firstly, it does not directly consider patient-paired CRLM and primary CRC, making it unclear how the molecular characteristics of the primary CRC are maintained after distant spread. While the mapping of CRC signatures suggests strong parallels, this remains an area requiring further clarification. On the other hand, the analysis of multiple CRLM based on the metagene HR-score suggests small intra-patient variability that indirectly implies a relatively homogeneous metastatic spread.

Secondly, our analysis is based on a cross-sectional cohort of bulk tumor samples without longitudinal follow-up data on tumor development over time. However, the observed cross-sectional heterogeneity can be interpreted in terms of a series of developmental CRLM states existing in the different patients along the pseudotime scale, which simulates developmental dynamics [44]. Alternatively, it could reflect the intra-tumoral CRLM heterogeneity of microscopic lesions all co-existing at the same stage of tumor progression. 

Both interpretations suggest that heterogeneous micro-lesions may change their composition along developmental trajectories of metastasis. This view is supported by spatial transcriptomics of CRLM, which show that cellular neighborhood archetypes reflecting different stages of tumor progression and resembling our LMS-types are active simultaneously in different spatial microregions of the same tumor [118]. Other studies have identified distinct modes of micro-vessel vascularization, specific metabolic alterations, and a *WNT*-signaling signature with a prognostic impact co-existing in the same CRLM [119]. Interestingly, a recent spatial transcriptomics study of synchronous resections of primary CRC and matched CRLM suggests that the stromal-versus-inflammatory balance of the invasive edge of CRLM samples impacts prognosis. Specifically, it reported that an increase in LMS5-like features (increased Tregs and stromal composition) compared with inflammatory ones is associated with a worse prognosis [120]. Another study along this line suggested that observable histopathological growth patterns (HGPs) can be interpreted in terms of molecular mechanisms [121]. These patterns differentiate between LMS5-like lesions (desmoplastic HGP, enriched in EMT, angiogenesis, stroma, and immune signatures) and lesions resembling LMS3-4 (replacement HGP, enriched in metabolism, cell cycle, and DNA damage repair signatures), as well as combinations of both. This implies that microscopic imaging in combination with spatial transcriptomics can support molecular diagnostics. Together, this data suggests that the heterogeneity of liver metastases appears to distribute on a microscopic scale, both spatially and temporally. This implies that different LMS-states and cellular TME-communities coexist in the same tumor and change their composition upon development, usually towards immune-suppressive and treatment-resistant states. Further investigation into these dynamics is necessary to fully understand their implications for treatment and prognosis.

## 5. Conclusions

Machine learning using an omics portrayal of CRLM provides a comprehensive and detailed understanding of the molecular heterogeneity underlying this complex disease. Our study revealed a shift from treatment-sensitive to treatment-resistant tumors that are guided by genetic, epigenetic, and microenvironmental factors. Our findings encourage further studies to better understand the micro-spatial heterogeneity of the CRLM, the underlying epigenetic mechanisms, the changing cellular communities in the TME interacting with the tumor cells, and also possible genetic determinants. Understanding this heterogeneity is crucial for tailoring effective treatment strategies, where precision medicine is one promising approach. It must involve the genomic profiling of individual tumors in follow-up settings to identify specific alterations and vulnerabilities in space and time to tackle the diverse manifestations of CRLM and provide better control of disease progression. This information can guide the selection of targeted therapies, immunotherapies, or combined treatments.

## Data Availability

An interactive online browser is provided, which provides different views on the CRLM-data set: https://apps.health-atlas.de/opossom-browser/?dataset=15 and www.izbi.de (see also Supplementary Figure S1).

## Supplementary Materials

The following supporting information can be downloaded at: https://www.mdpi.com/article/10.3390/cancers15153835/s1, Figure S1: Browsing the livermets-dataset using the oposSOM-browser; Figure S2. Gallery of SOM portraits in standard and coastline scales; Figure S3: Sorting the portraits; Figure S4: Heatmaps of the gene set categories gene ontology biological process (GO BP) and immunome (taken from [4]); Figure S5: Detailed functional analysis using gene set maps and profiles; Figure S6: Single cell analysis of CRLM data for cell marker gene extraction and mapping to CRLM data; Figure S7. Pathway activity analysis of *WNT*-signaling; Figure S8: Pseudo-dynamics analysis of CRLM development; Figure S9: Heterogeneity of CAF and immune cell expression across the LMS; Figure S10. Comparison of different segmentation methods for visualizing the expression land-scape, extracting expression spot-modules and characterizing them in terms of spot implications and mutual correlations between them; Table S1. Spot module characteristics; Table S2: is provided as excel sheet. It contains lists of spot genes A-F; Table S3: Collection of gene sets specifically implemented in oposSOM in this publication; Table S4: is provided as supplementary excel sheet. List of genes with maximum and minimum HR, list of delta-HR ranked CRLM. See also Figure 7c. References [122,123,124,125,126,127,128,129,130] are cited in the supplementary materials. Movie S1: Tree-trajectory-Seg1-to-Seg3.

## Abbreviations

ALT	Alternative lengthening of telomeres
AUC	Area Under the ROC Curve
CAF	Cancer-associated fibroblast
CIN	Chromosomal instability
CRC	Colorectal cancer
CRLM	Colorectal liver metastases
CMS	Consensus molecular subtyping
CNV	Copy number variations
CIMP	CPG island methylation phenotype
EPC	Endothelial progenitor cell
ECM	Extracellular matrix
FU	Fluorouracil
GM	Gene module
GO	Gene ontology
GINS	Gene-interaction-perturbation-network-based subtyping
GPCR	G-protein coupled receptor
HR	Hazard ratio
HepSC	Hepatic stellate cell
HGP	Histopathological growth patterns
ICI	Immune checkpoint inhibitor
ICA	Independent component analysis
KEGG	Kyoto Encyclopedia of Genes and Genomes
LIV	Liver
LM	Liver metastases
LMS	Liver metastasis subtype
LSEC	Liver sinusoidal endothelial cell
MSI	Microsatellite instable
NK	Natural killer
OS	Overall survival
OC	lOverlap coefficient
PSF	Pathway signal flow
PC	Plasma cells
pDC	Plasmacytoid dendritic cells
PRC2	Polycomb repressive complex 2
PCA	Principal component analysis
PT	Pseudotime
ROC	Receiver operator characteristic
SOM	Self-organizing map
scRNAseq	Single cell RNA sequencing
TAF	Tumor associated macrophages
TEL	Telomerase
TM	Telomere maintenance
TCGA	The cancer genome atlas
TF	Transcription factor
TME	Tumor microenvironment
UMAP	Uniform manifold approximation and projection for dimension reduction
UMI	Unique molecular identifier
WTO	Weighted topological overlap
