# Supplementary material for: Transcriptomic Maps of Colorectal Liver Metastasis: Machine Learning of Gene Activation Patterns and Epigenetic Trajectories in Support of Precision Medicine
Source: Cancers (Basel). 2023 Jul 28;15(15):3835. doi: 10.3390/cancers15153835 (PMC10417131; doi:10.3390/cancers15153835)
Supplement: Supplementary file 1 [file cancers-15-03835-s001.zip › Supplementary_Figures_and_Table_S1.pdf]

Article

Transcriptomic Maps of Colorectal Liver Metastasis: Machine Learning of Gene Activation Patterns and Epigenetic Trajectories in Support of Precision Medicine

Ohanes Ashekyan [1,†], Nerses Shahbazyan [1,†], Yeva Bareghamyan [1], Anna Kudryavzeva [1], Daria Mandel [1], Maria Schmidt [2], Henry Loeffler-Wirth [2], Mohamed Uduman [3], Dhan Chand [3], Dennis Underwood [3], Garo Armen [3], Arsen Arakelyan [4], Lilit Nersisyan [1] and Hans Binder [1,2,*]

[1] Armenian Bioinformatics Institute, 3/6 Nelson Stepanyan Str., Yerevan 0062, Armenia; ohanes.ashekyan@abi.am (O.A.); nerses.shahbazyan@abi.am (N.S.); yeva.bareghamyan@abi.am (Y.B.); anyakudryavceva804@gmail.com (A.K.); daria.laricheva@abi.am (D.M.); lilit.nersisyan@abi.am (L.N.)
[2] IZBI, Interdisciplinary Centre for Bioinformatics, Universität Leipzig, Härtelstr. 16–18, 04107 Leipzig, Germany; schmidt@izbi.uni-leipzig.de (M.S.); wirth@izbi.uni-leipzig.de (H.L.-W.)
[3] Agenus Inc., 3 Forbes Road, Lexington, MA 7305, USA; mohamed.uduman@agenusbio.com (M.U.); dhan.chand@agenusbio.com (D.C.); dennis.underwood@agenusbio.com (D.U.); armen@agenusbio.com (G.A.)
[4] Institute of Molecular Biology of the National Academy of Sciences of the Republic of Armenia, 7 Has-Ratyan Str., Yerevan 0014, Armenia; arsen.arakelyan@abi.am
* Correspondence: hans.binder@abi.am
† These authors contributed equally to this work.

Supplementary Figures and Table S1

Appendix A (supplementary Figures and supplementary Table S1)

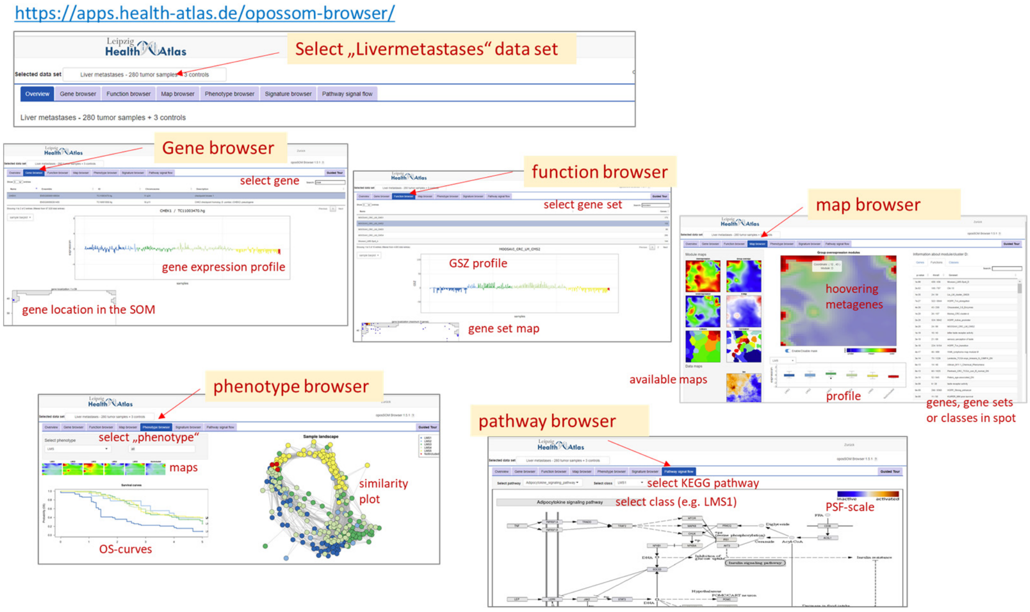

Supplementary Figure S1. Browsing the livermets-dataset using the oposSOM-browser: (i) Select the ”livermetastases” data set (loading takes about a minute); (ii) Choose “gene browser” to select a gene and view its expression profile and position in the map; (ii) choose “function browser” to select a gene set and view its GSZ-profile and gene map; (iii) Choose “map browser” to select a map-type (e.g. mean group maps, prognostic map etc.), hoover to select a metagene/spot and check-out genes, gene sets of group assignment; (iv) choose “phenotype browser” and select an item (LMS, sex, *KRAS* mutation etc.) and check-out mean maps, OS-curves and similarity net; (v) choose “Pathway browser” and select the Kyoto encyclopedia of genes and genomes (KEGG)-pathway to check-out the pathway signal flow (PSF) activity status of the node-genes in a LMS-specific fashion (see [1] for methods description).

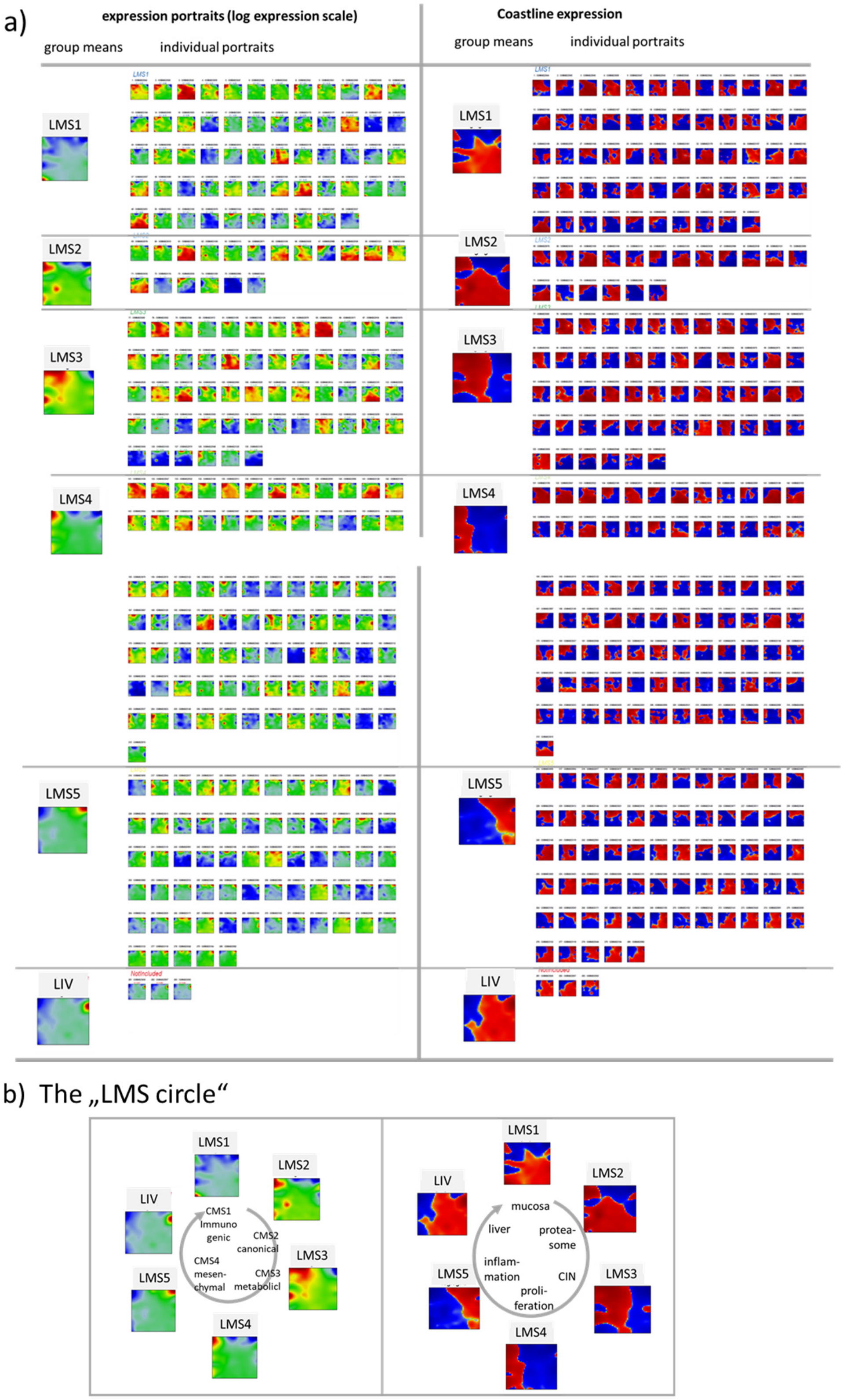

Supplementary Figure S2. Gallery of SOM portraits in standard and coastline scales. a) Individual SOM-portraits of the transcriptomes of 283 CRLM taken from [2]. Two color scales were used, standard scale color codes log(expression) from minimum (blue) and maximum (red) values in each portrait. The coastline scale uses the mean expression of each metagene averaged over all samples as “sea-level”. It is red for metagene expression exceeding this mean (“land-areas”) and blue for smaller values (“sea areas”. b) The red overexpression regions and spots rotate in clockwise

direction in the LMS-stratified mean portraits thus indicating mutual similarities and possibly developmental relatedness. The standard portraits (left part) resolve the spot-regions of strong over- and under-expression in red and blue, respectively. The coastline portraits resolve the areas of weak over- (red) and under- (blue) expression. The different LMS-portraits mutually overlap thus indicating possible transitions between them via the shift of the associated molecular functions.

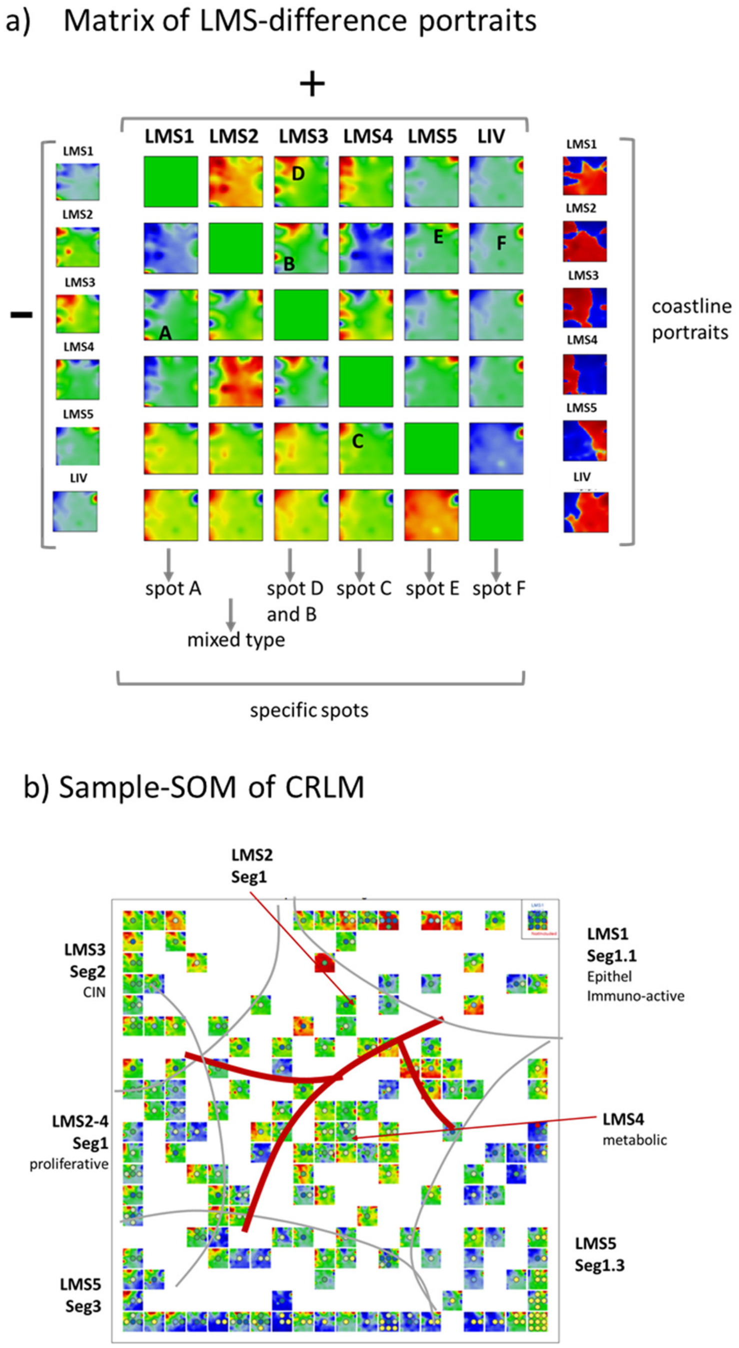

Supplementary Figure S3. Sorting the portraits. a) Difference portraits between the subtypes reveal subtype specific spots and expression patterns (see arrows below). One sees, e.g., that LMS3 shows specific activation of spot D (CIN) and B, while LMS4 overexpresses spot C (proliferation) and, to different degrees, the triple-spots at the left edge of the difference map LMS4- LMS3 relates to metabolic functions (e.g. oxphos). The mixed type LMS2 combines features of LMS1 and LMS5 as well as of LMS3 and 4 b) The sample-SOM (or 2nd level SOM) provides a two-dimensional similarity map of the individual portraits [3]. Different areas enrich different LMS (grey-borders) and also reflect

roughly the monocle-tree structure (segments) discussed in the paper (red branches). Note that archetypes of the LMS distribute along the edges of the map while mixed and/or transition types accumulate in its central part.

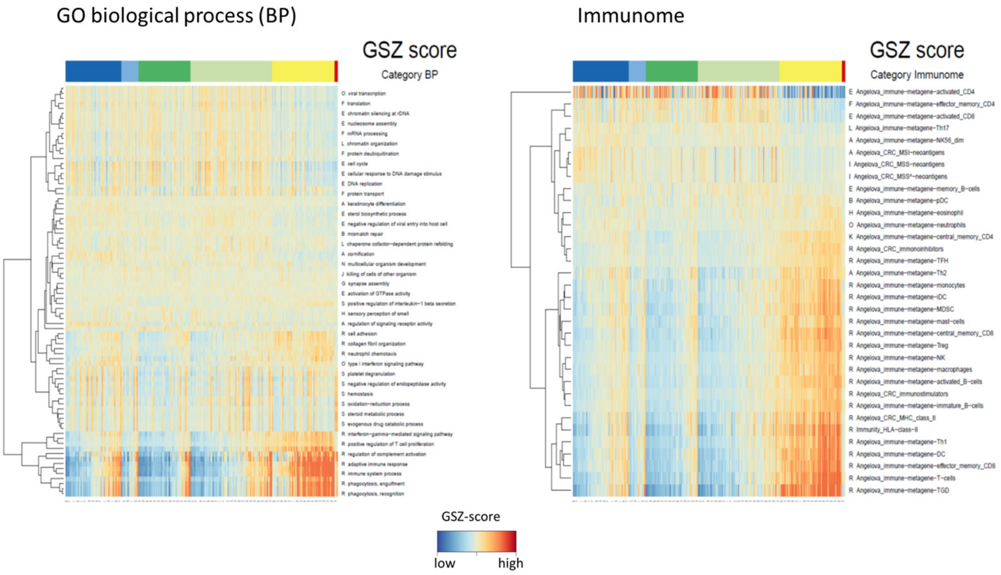

Supplementary Figure S4. Heatmaps of the gene set categories gene ontology biological process (GO BP) and immunome (taken from [4]).

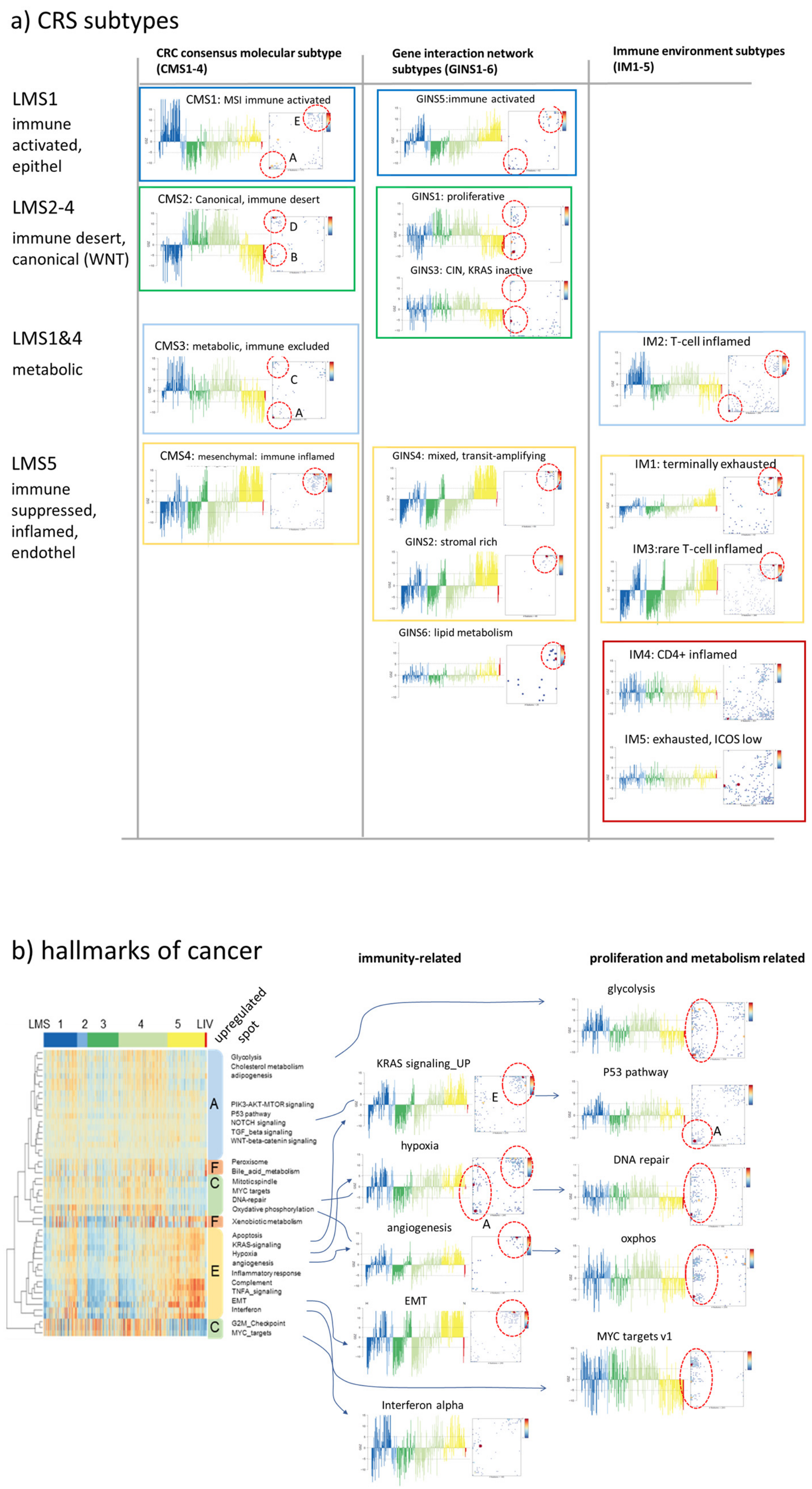
a) CRS subtypes
CRC consensus molecular subtype (CMS1-4)
Gene interaction network subtypes (GINS1-6)
Immune environment subtypes (IM1-5)
LMS1 immune activated, epithel
LMS2-4 immune desert, canonical (WNT)
LMS1&4 metabolic
LMS5 immune suppressed, inflamed, endothel
CMS1: MSI immune activated
GINS5:immune activated
CMS2: Canonical, immune desert
GINS1: proliferative
GINS3: CIN, KRAS inactive
CMS3: metabolic, immune excluded
IM2: T-cell inflamed
CMS4: mesenchymal: immune inflamed
GINS4: mixed, transit-amplifying
IM1: terminally exhausted
GINS2: stromal rich
IM3:rare T-cell inflamed
GINS6: lipid metabolism
IM4: CD4+ inflamed
IM5: exhausted, ICOS low
b) hallmarks of cancer
immunity-related
proliferation and metabolism related
LMS 1 2 3 4 5 LIV
upregulated spot
Glycolysis
Cholesterol metabolism
adipogenesis
PIK3-AKT-MTOR signaling
P53 pathway
NOTCH signaling
TGF_beta signaling
WNT-beta-catenin signaling
Peroxisome
Bile_acid_metabolism
Mitoticspindle
MYC targets
DNA-repair
Oxydative phosphorylation
Xenobiotic metabolism
Apoptosis
KRAS-signaling
Hypoxia
angiogenesis
Inflammatory response
Complement
TNFA_signaling
EMT
Interferon
G2M_Checkpoint
MYC_targets
KRAS signaling_UP
hypoxia
angiogenesis
EMT
Interferon alpha
glycolysis
P53 pathway
DNA repair
oxphos
MYC targets v1

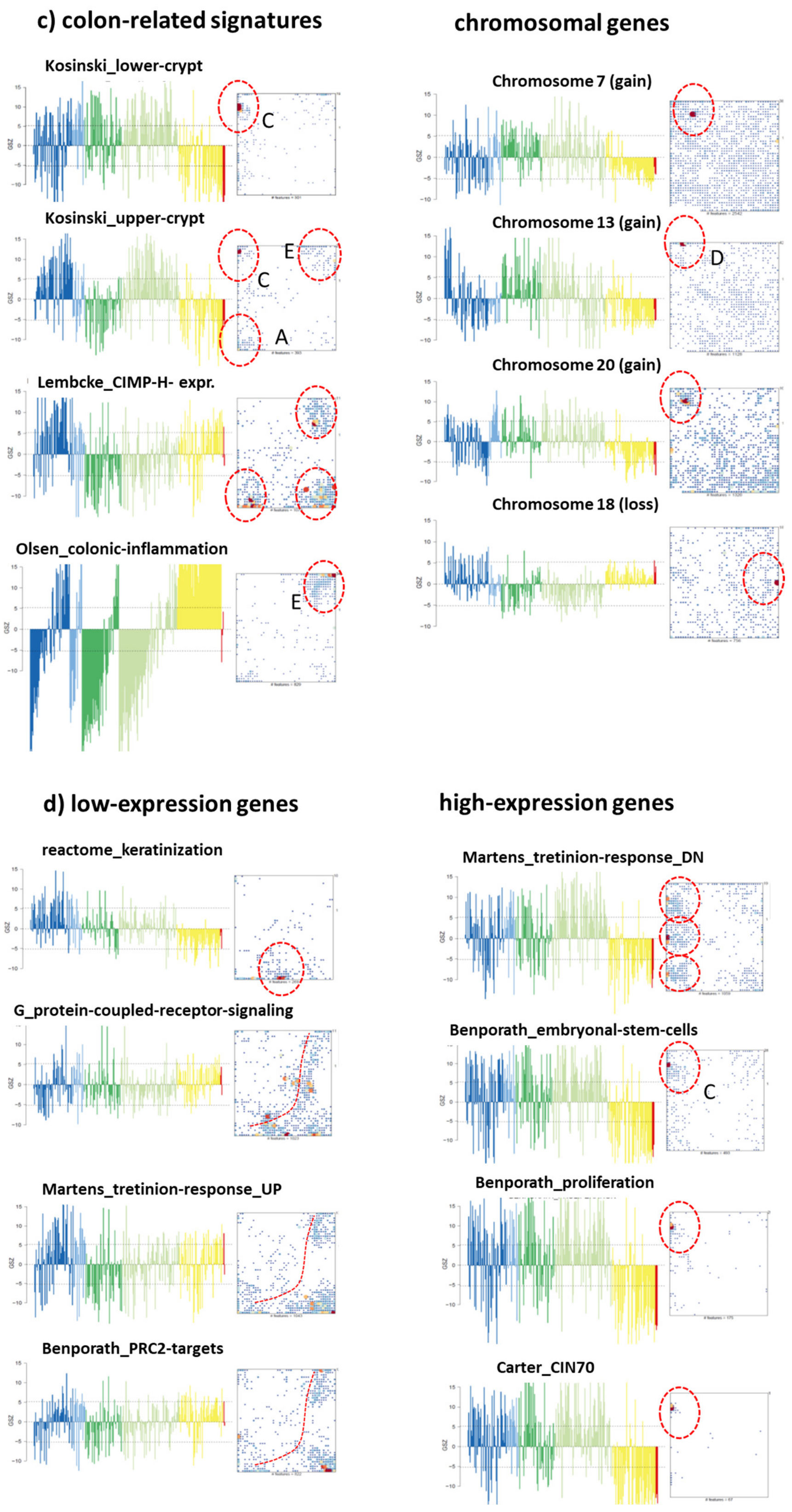
c) colon-related signatures
chromosomal genes
Kosinski_lower-crypt
C
Kosinski_upper-crypt
E
C
A
Lembcke_CIMP-H- expr.
Olsen_colonic-inflammation
E
Chromosome 7 (gain)
Chromosome 13 (gain)
D
Chromosome 20 (gain)
Chromosome 18 (loss)
d) low-expression genes
high-expression genes
reactome_keratinization
G_protein-coupled-receptor-signaling
Martens_tretinion-response_UP
Benporath_PRC2-targets
Martens_tretinion-response_DN
Benporath_embryonal-stem-cells
C
Benporath_proliferation
Carter_CIN70
GSZ

e) chromatin states

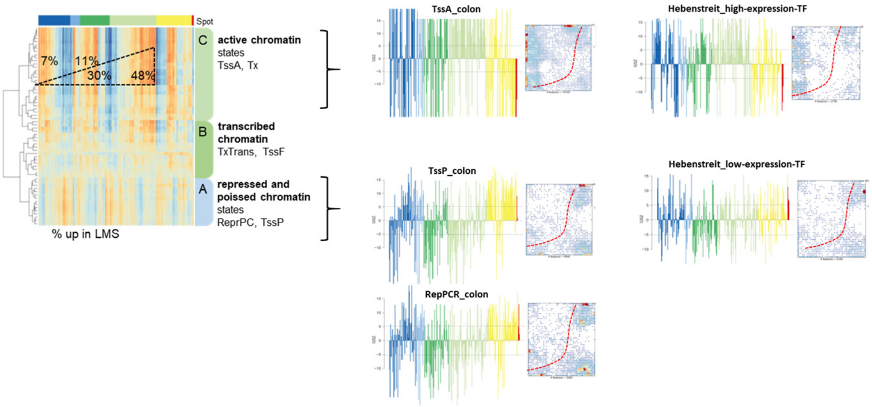

Supplementary Figure S5. Detailed functional analysis using gene set maps and profiles: The profiles show the activation status across all samples in units of the gene set Z-score (GSZ). The GSZ value is calculated as the mean centralized expression averaged over the set-genes divided by its variance over all samples. CRLM were sorted and color coded as in the main paper. The gene set map shows the distribution of the genes of the set in the SOM which reflects their topological impact in the expression (co-variance) landscape. Often genes accumulate in and around the spot regions (red dashed circles) what assigns the functional context of the respective areas in the SOM and supports interpretation.

a) Signature sets of CRC subtypes were sorted according to their resemblance with LMS. CRC subtypes and signatures were taken from [5, 6, 7] (see **Figure 3**b-d). IM4 and IM5 resemble low-expression TF characteristics (see below).

b) Hallmarks of cancer signatures [8] roughly split into immunity-related and proliferation and metabolism-related hallmarks. Genes of the former category accumulate in/around spots E and/or A while the latter ones are found along the left edge of the map. See also **Figure 3**i.

c) Colon and CRC related signatures refer to upper and lower crypt expression in the healthy colon [9] revealing more metabolic and proliferative activity, respectively; genes overexpressed in CIMP-high (CpG island methylator phenotype) CRC referring to hypomethylated DNA-promoters [10] resembling the patterns of PRC2-targets (see below) and of inflamed colon signature (inflamed (ulcerative colitis)-versus-not inflamed) [11]. The right part shows the profiles and maps of genes located at chromosomes showing gains or losses in CRC and CRLM. Gained genes accumulate in/near spot D upregulated in LMS3.

d) Gene signatures of low and high expression categories accumulate in the right and left parts, respectively. The former ones include the "plasticity" plateau in the right lower corner. Proliferation-related signatures include that of embryonal stem cells [12] and of CIN (chromosomal instability) [13] which can associate with bad and good prognosis in the oligometastatic context [14, 15].

e) Genes referring to active and repressive/poised chromatin states in the healthy colon [16] accumulate in the left and right part of the map, respectively, and thus associate with functions shown in part d.

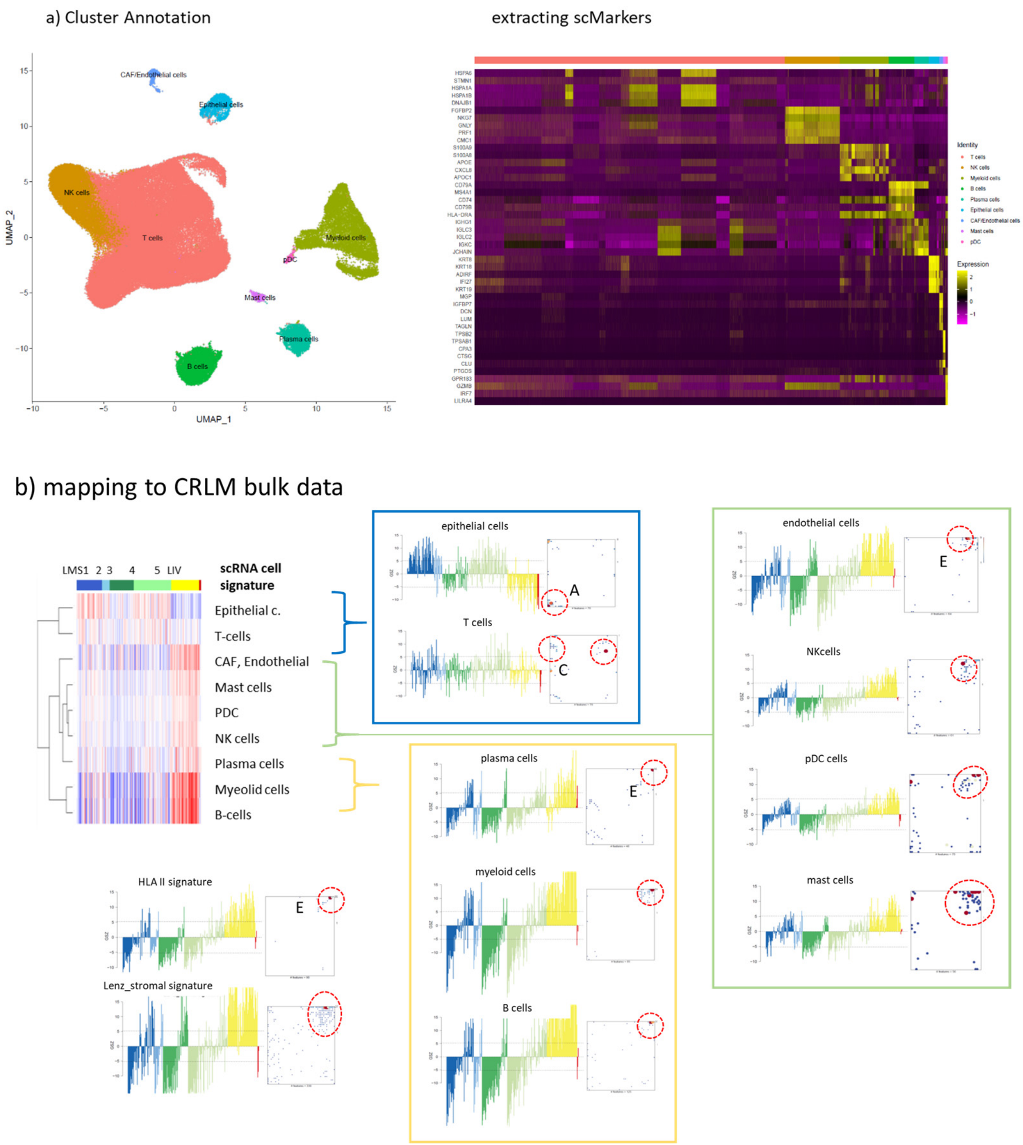

Supplementary Figure S6. Single cell analysis of CRLM data for cell marker gene extraction and mapping to CRLM data: a) UMAP (Uniform Manifold Approximation and Projection for Dimension Reduction) plot of single cell transcriptomics (scRNA) analysis of CRLM data taken from [17] resolves different cell-types populating the TME. b) Marker genes of the different cell types extracted form scRNA data analysis. b) Single-cell markers taken from the scRNAseq analysis of CRLM.

scRNAseq analysis: Preprocessed data was used as an input to construct a Seurat object using the Seurat scRNA-seq analysis R package [18]. After quality control, 25,121 protein coding genes and 11,292 single cells were considered for downstream analysis. Raw unique molecular identifier (UMI) counts were log-normalized using the scale of 10,000. Top 2,000 highly variable genes were identified to be used as distinctive genes in downstream analysis. “Var.to.regress” option UMI’s and percent mitochondrial content were used to regress out unwanted sources of variation. Linear dimensionality reduction i.e. principal component analysis (PCA) was run and the dimensionality of the datasets was identified as 10 principal components. Then, “FindClusters” was run on 10 principle components with 0.5 resolution to perform clustering and annotation. As described in Che et al. [17] the annotation of each cell cluster was confirmed by the expression of canonical marker genes. We used the following genes advised for cluster annotation [17]: EPCs were identified using the higher expression of EPCAM, and other cell types, which were annotated using T cells (*CD3D, CD3G, TRAC*), B cells (*CD19, CD79A, and MS4A1*), plasma cells, monocytes and macrophages (*CD68, CD163, CD14, and LYZ*), NK Cells (*KLRF1, KLRD1, FGFBP2, and PRF1*). CAFs (*FAP, COL1A1, COL3A1, DCN, and ACTA2*), endothelial cells (*CLDN5, CDH5, and VMF*), pDC (*LILRA4 and IL3RA*),

and mast cells (*TPSAB1, TPSB2, and MS4A2*). Also, FindAllMarkers was run to extract distinctive markers for each major cellular cluster to be used as CRLM-characterizing gene signatures.

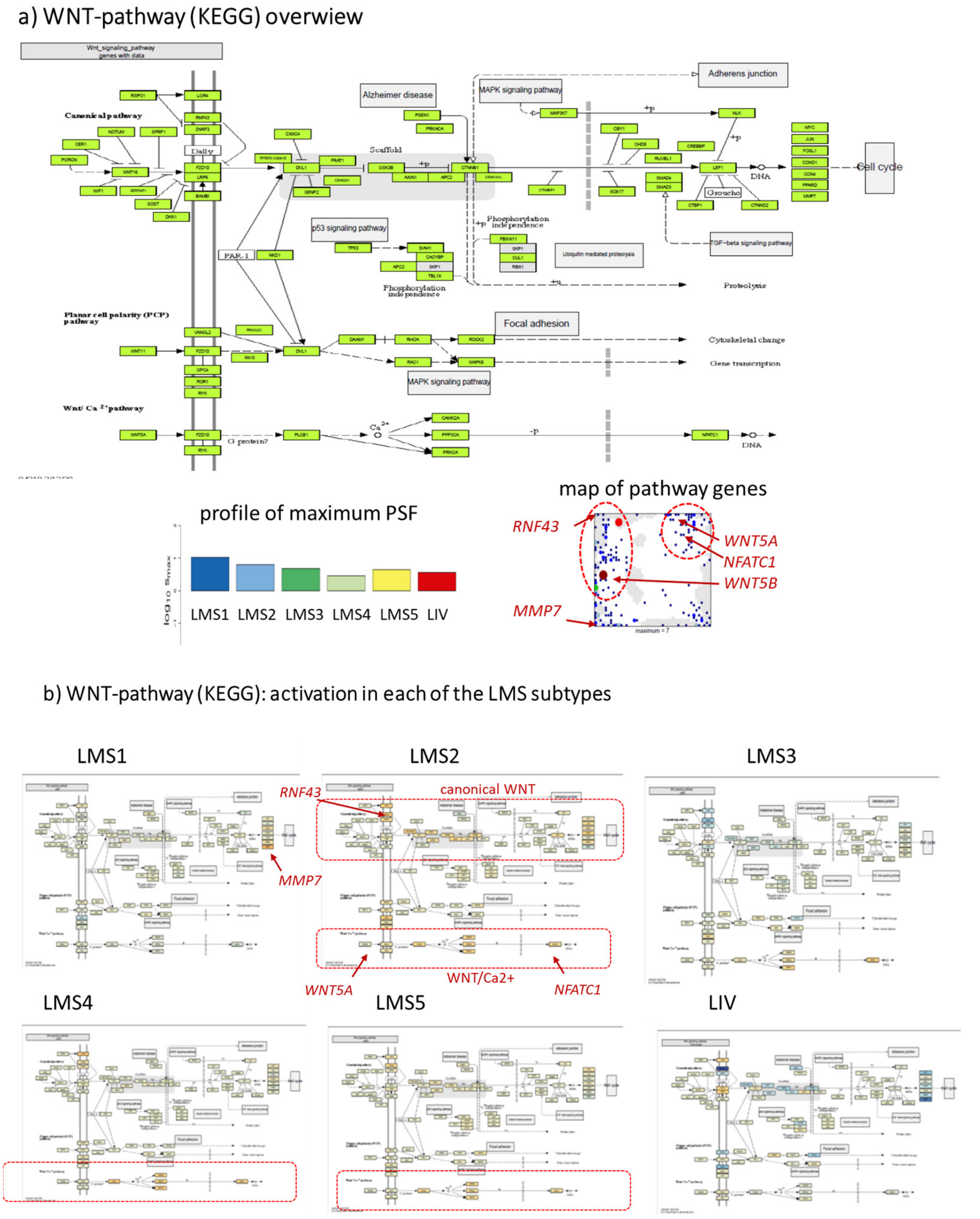

Supplementary Figure S7. Pathway activity analysis of *WNT*-signaling. a) Pathway topology, maximum pathway signal flow (PSF) in the different LMS and map of the pathway genes. Genes of interest (see b) locate in/near spot A (*MMP7*), C (*RNF43*) and E(*WNT5A*) b) Subtype-specific activation patterns show high PSF-value of MMP7, one of the sink nodes of canonical WNT in LMS1. *MMP7* locates in spot A which associates with epithelial function and canonical WNT activation. In LMS2 one finds activation of both, the canonical pathway including also *RNF43* and the non-canonical WNT/CA2+ signaling associated with activation of *WNT5A* and of the sink *NFATC1*, both located around spot E associated with endothelial function. *MMP7* is reported to be involved in CRC metastasis [19] while *WNT5A* is reported to promote EMT and aggressive and proliferative

phenotypes in CRC [20] and other cancer types [21]. The ubiquitin ligase coding gene *RNF43* is frequently mutated in CRC [22].

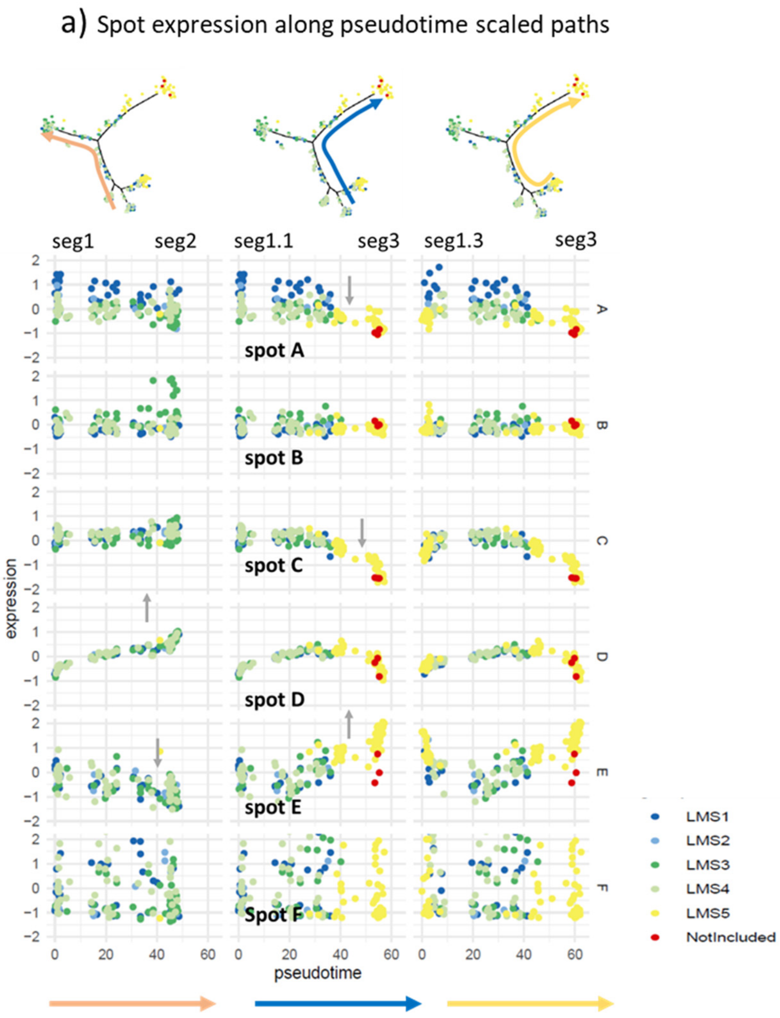

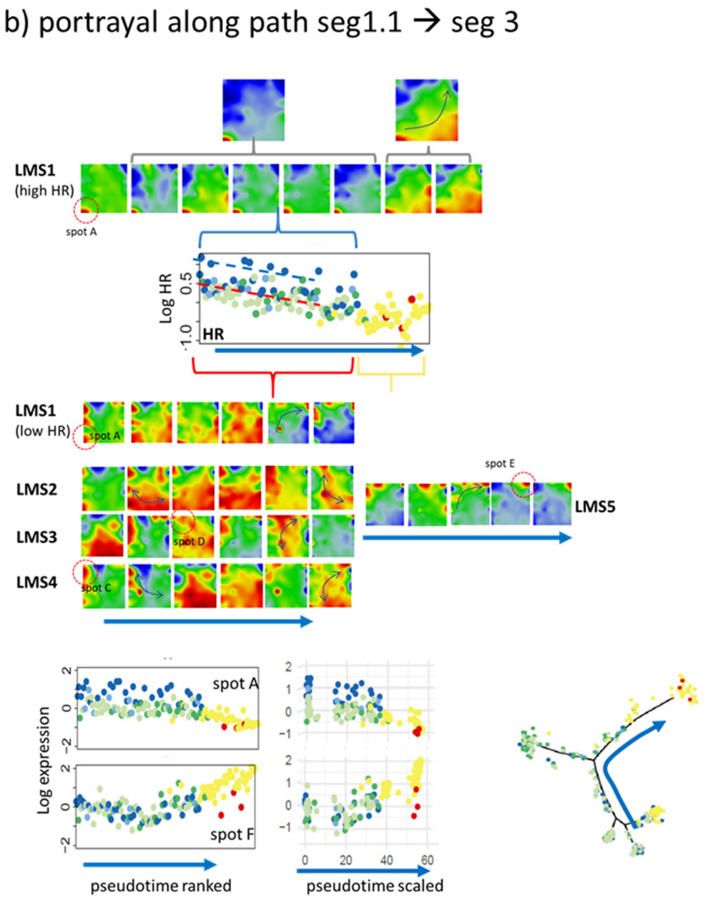

Supplementary Figure S8. Pseudo-dynamics analysis of CRLM development. a) Expression profiles of spots A-F along three trajectories along the monocle-tree in pseudotime-similarity scale. The specifics of the trajectories is determined mainly by different slopes of spots C- E. b) Portrayal of CRLM along trajectory seg1.1→ 3 (blue arrow) reveals details of the pseudo-dynamics of changes in the expression landscape. CRLM from LMS1 split into high HR (inferior prognosis, blue dashed line) and low HR (better prognosis), both decaying along the trajectory, and associating with high and lower expression of spot A. High HR portraits show a shift of expression from spot A via the plasticity plateak towards spot E while low HR portraits of LMS1 mix with spot pattern seen in LMS2-4 and the plasticity plateau as well also towards spot E. CRLM of LMS2-4 found along the trajectory show specific high expression of the respective marker spot but similar patterns including the plasticity plateau. Overall we see an interplay between expression patterns of the high-expression spots and the low-expression regions of epigenetic context where in direction towards the split point towards Seg3 the red overexpression patterns shift towards spot E.

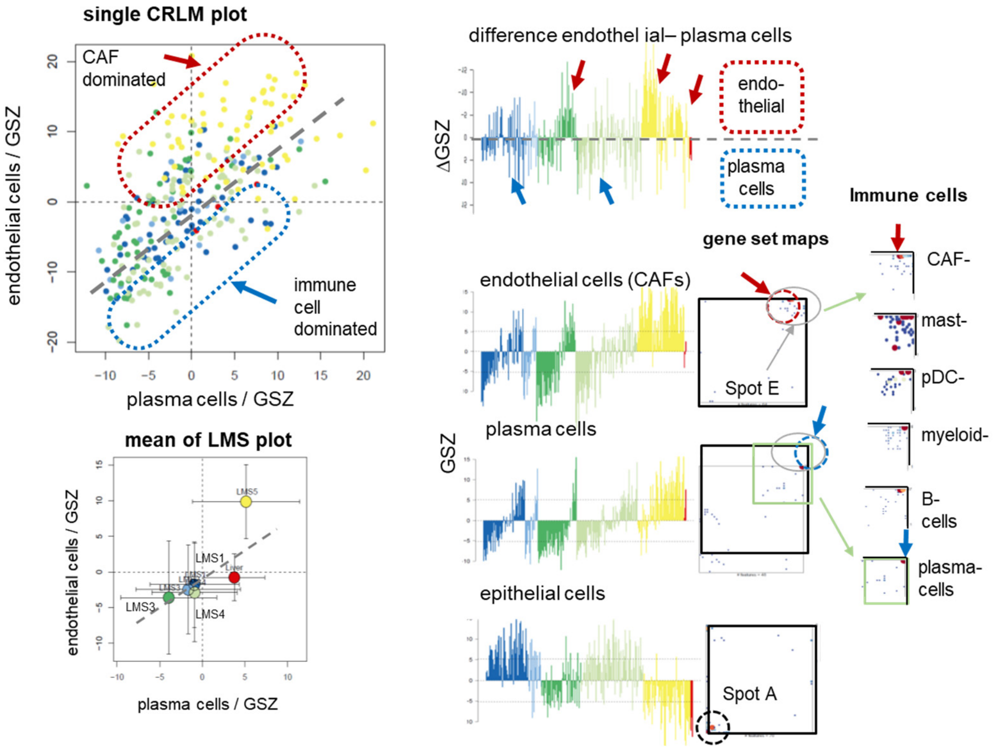

Supplementary Figure S9. Heterogeneity of CAF and immune cell expression across the LMS. Spot E associates with the TME which contains endothelial/CAF and inflammatory cells. The biplot of both compounds (where we selected plasma cells (PC) as a proxy of the immune compound) shows an overall linear relation between them with maximal values in LMS5 (left part). The individual CRLM-values considerably scatter with large deviations into positive (CAF dominance, red dashed frame and arrow) and negative (PC dominance, blue dashed frame and arrow) directions, especially for LMS5 (yellow dots) and LMS4 (light green), respectively. These variations reflect relative PC-dominance in LMS1 and CAF dominance in LMS5, and, in general, varying CAF-contributions across the LMS (right part). In the map, CAF marker genes locate more in the left part of spot E while PC markers are slightly shifted towards its right part closer to the upper corner. Marker genes of the other immune cells show that B-cells distribute similarly to PC while mast cells more resemble the distribution of CAFs which overall reflects a CRLM-specific change of cell communities of the TME. Comparison of the portraits of the LMS5 tumors in Seg1.3 and Seg.3 show that they refer to a more endothel/CAF enriched and depleted TME, respectively (see main paper, **Figure 4** and Supplementary Figure 6b).

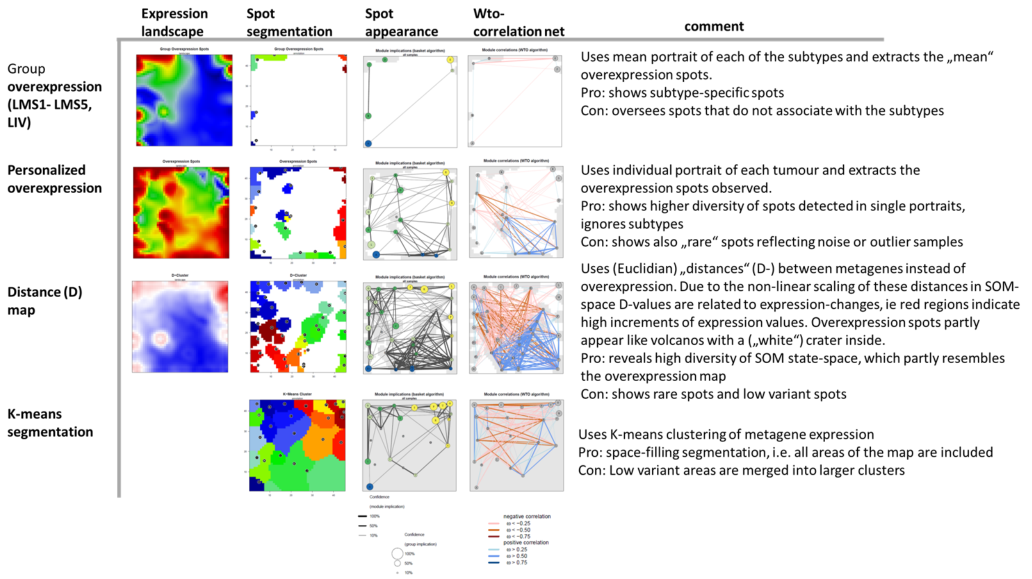

Supplementary Figure S10. Comparison of different segmentation methods for visualizing the expression landscape, extracting expression spot-modules and characterizing them in terms of spot implications and mutual correlations between them. The weighted topological overlap correlation and spot implication (joint appearance of spots) has been described previously in [23] and [24], respectively. See comments in the figure for details.

Supplementary Table S1. Spot module characteristics.

Spot	Brief characteristics	Up/DN	Top genes a)	enriched gene sets b)
A	epithelium, CMS1-resemblance	Up in LMS1 DN in LMS5	*GCNT3, GJB3, TSPAN1, LIPH, EPHA2*	Lembcke_CIMP-CRC-UP (-39), Sabates_Colorectal_Adenoma_UP (-29), Jaeger_Metastasis_DN (-26), Wirth_Mucosa (-21), Wu_Cell-migration (-18), Kosinsky_top-crypt (-16), Bild_HRAS-oncogenic-signature (-16), Moosavi_CMS1 (-16), Marisa_CRC-clusterf (-15)
B	metabolism, PRC2-targets	Up in LMS3	*TRPM3, PLPPR1, GALNT13, OSBPL6, ADAMTS18*	Lembcke_TCGA-expr_kmeans_M_CIMP.H_DN(-41), CHIANG_LIVER_CANCER_SUBCLASS_CTNNB1_UP(-23), HOPP_Repressed(-20), HOPP_Poised_Promoter(-15), SABATES_COLORECTAL_ADENOMA_UP(-15), Lembcke_TCGA-expr_kmeans_L_CIMP.H_UP_Cluster4_DN(-14), integral component of membrane(-14), MEISSNER_BRAIN_HCP_WITH_ H3K4ME3_AND_H3K27ME3(-11), Xie_Senescence (-9), Benporath:Suz21-targets (-9)
C	Proliferation, cell cycling	Up in LMS1-LMS4; DN in LMS5	*TPX2, MYB, GALNT3, ECT2, ESRP1*	OTIRIOU_BREAST_CANCER_GRADE_1_VS_3_UP(-99), Liu_LM_cluster_GM16(-99), Wirth_lymphoma map module D(-99), WILLSCHER_GBM_Verhaak-CL_up(-99), Gerber_wt/wt_melanoma-cells-SpotA(-99), Hopp_blood module K(-99), FISCHER_DREAM_TARGETS(-99), FLORIO_NEOCORTEX_BASALRADIAL_GLIA_DN(-99), MEBARKI_HCC_PROGENITORFZD8CRD_UP(-99), REACTOME_CELL_CYCLE(-99)
D	CIN, CMS2-resembling	Up in LMS3	*ZFP64, TIA1, RBM39, BCL11A, SMG1*	Chr13(-52), Liu_LM_cluster_GM26(-35), HOPP_Txn_elongation(-27), Chaussabel_3,8_Enzymes(-26), Marisa_CRC_cluster-d(-20), HOPP_Active_promoter(-20), MOOSAVI_CRC_LM_CMS2(-20), bitter taste receptor activity(-19), sensory perception of taste(-19), HOPP_Txn_transiton(-18)
E	Immune response, stroma	Up in LMS5	*PIK3CG, PTPRC, ARHGEF6, CD53, NCKAP1L*	adaptive immune response (-99), antigen binding(-99), B cell recpetor signaling pathway(-99), complement activation classical pathway(-99), defense response to bacterium(-99), external side of plasma membrane(-99), extracellular region(-99), immune response(-99) , immune system process(-99), immunoglobulin complex(-99)
F	Liver		*PLG, AMBP, SLC2A2, C8A, ITIH1*	Wirth_Liver(-99), Liu_LM_cluster_GM17(-99), HSIAO_LIVER_SPECIFIC_GENES(-99), blood microparticle(-83), CHIANG_LIVER_CANCER

a) Top 5 genes with largest Pearsons-correlation coefficient regarding the mean spot profile, full lists for each spot are provided in Supplementary Table 2

b) Top enriched gene sets (Fishers exact test, exponent of p-value is given in the brackets) taken from the repository implemented in oposSOM

Supplementary Table S2: is provided as excel sheet. It contains lists of spot genes A-F.

Supplementary Table S3: Collection of gene sets specifically implemented in oposSOM in this publication.

Supplementary Table S4: is provided as supplementary excel sheet. List of genes with maximum and minimum HR, list of delta-HR ranked CRLM. See also Figure 7c.

References

1. Nersisyan, L.; Loeffler-Wirth, H.; Arakelyan, A.; Binder, H., Gene set- and pathway- centered knowledge discovery assigns transcriptional activation patterns in brain, blood and colon cancer - A bioinformatics perspective. *Journal of Bioinformatics Knowledge Mining* **2016,** 4, (2), 46-70.
2. Moosavi, S. H.; Eide, P. W.; Eilertsen, I. A.; Brunsell, T. H.; Berg, K. C. G.; Røsok, B. I., . . . Sveen, A., De novo transcriptomic subtyping of colorectal cancer liver metastases in the context of tumor heterogeneity. *Genome Medicine* **2021,** 13, (1), 143.
3. Wirth, H.; Löffler, M.; von Bergen, M.; Binder, H., Expression cartography of human tissues using self organizing maps. *BMC Bioinformatics* **2011,** 12, (1), 306.
4. Angelova, M.; Charoentong, P.; Hackl, H.; Fischer, M. L.; Snajder, R.; Krogsdam, A. M., . . . Trajanoski, Z., Characterization of the immunophenotypes and antigenomes of colorectal cancers reveals distinct tumor escape mechanisms and novel targets for immunotherapy. *Genome Biology* **2015,** 16, (1), 1-17.
5. Guinney, J.; Dienstmann, R.; Wang, X.; de Reynies, A.; Schlicker, A.; Soneson, C., . . . Tejpar, S., The consensus molecular subtypes of colorectal cancer. *Nat Med* **2015,** 21, (11), 1350-1356.
6. Liu, Z.; Weng, S.; Dang, Q.; Xu, H.; Ren, Y.; Guo, C., . . . Han, X., Gene interaction perturbation network deciphers a high-resolution taxonomy in colorectal cancer. *eLife* **2022,** 11, e81114.
7. Yang, S.; Qian, L.; Li, Z.; Li, Y.; Bai, J.; Zheng, B., . . . Chen, L., Integrated Multi-Omics Landscape of Liver Metastases. *Gastroenterology* **2023,** 164, (3), 407-423.e17.
8. Liberzon, A.; Birger, C.; Thorvaldsdóttir, H.; Ghandi, M.; Mesirov, Jill P.; Tamayo, P., The Molecular Signatures Database Hallmark Gene Set Collection. *Cell Systems* **2015,** 1, (6), 417-425.
9. Kosinski, C.; Li, V. S. W.; Chan, A. S. Y.; Zhang, J.; Ho, C.; Tsui, W. Y., . . . Chen, X., Gene expression patterns of human colon tops and basal crypts and BMP antagonists as intestinal stem cell niche factors. *Proceedings of the National Academy of Sciences* **2007,** 104, (39), 15418-15423.
10. Binder, H.; Hopp, L.; Lembcke, K.; Wirth, H., Personalized Disease Phenotypes from Massive OMICs Data. In *Big Data Analytics in Bioinformatics and Healthcare*, Baoying, W.; Ruowang, L.; William, P., Eds. IGI Global: Hershey, PA, USA, 2015; pp 359-378.
11. Olsen, J.; Gerds, T. A.; Seidelin, J. B.; Csillag, C.; Bjerrum, J. T.; Troelsen, J. T.; Nielsen, O. H., Diagnosis of ulcerative colitis before onset of inflammation by multivariate modeling of genome-wide gene expression data. *Inflammatory Bowel Diseases* **2009,** 15, (7), 1032-1038 10.1002/ibd.20879.

12. Ben-Porath, I.; Thomson, M. W.; Carey, V. J.; Ge, R.; Bell, G. W.; Regev, A.; Weinberg, R. A., An embryonic stem cell-like gene expression signature in poorly differentiated aggressive human tumors. *Nat Genet* **2008,** 40, (5), 499-507.

13. Carter, S. L.; Eklund, A. C.; Kohane, I. S.; Harris, L. N.; Szallasi, Z., A signature of chromosomal instability inferred from gene expression profiles predicts clinical outcome in multiple human cancers. *Nat Genet* **2006,** 38, (9), 1043-8.

14. Spurr, L. F.; Martinez, C. A.; Katipally, R. R.; Iyer, S. C.; Pugh, S. A.; Bridgewater, J. A., . . . Pitroda, S. P., A proliferative subtype of colorectal liver metastases exhibits hypersensitivity to cytotoxic chemotherapy. *npj Precision Oncology* **2022,** 6, (1), 72.

15. Pitroda, S. P.; Khodarev, N. N.; Huang, L.; Uppal, A.; Wightman, S. C.; Ganai, S., . . . Weichselbaum, R. R., Integrated molecular subtyping defines a curable oligometastatic state in colorectal liver metastasis. *Nat Commun* **2018,** 9, (1), 1793.

16. Ernst, J.; Kellis, M., Chromatin-state discovery and genome annotation with ChromHMM. *Nature Protocols* **2017,** 12, (12), 2478-2492.

17. Che, L.-H.; Liu, J.-W.; Huo, J.-P.; Luo, R.; Xu, R.-M.; He, C., . . . Li, J.-M., A single-cell atlas of liver metastases of colorectal cancer reveals reprogramming of the tumor microenvironment in response to preoperative chemotherapy. *Cell Discovery* **2021,** 7, (1), 80.

18. Hao, Y.; Hao, S.; Andersen-Nissen, E.; Mauck, W. M., 3rd; Zheng, S.; Butler, A., . . . Satija, R., Integrated analysis of multimodal single-cell data. *Cell* **2021,** 184, (13), 3573-3587.e29.

19. Zeng, Z. S.; Shu, W. P.; Cohen, A. M.; Guillem, J. G., Matrix metalloproteinase-7 expression in colorectal cancer liver metastases: evidence for involvement of MMP-7 activation in human cancer metastases. *Clin Cancer Res* **2002,** 8, (1), 144-8.

20. Bakker, E. R. M.; Das, A. M.; Helvensteijn, W.; Franken, P. F.; Swagemakers, S.; van der Valk, M. A., . . . Smits, R., Wnt5a promotes human colon cancer cell migration and invasion but does not augment intestinal tumorigenesis in Apc 1638N mice. *Carcinogenesis* **2013,** 34, (11), 2629-2638.

21. Wang, B.; Tang, Z.; Gong, H.; Zhu, L.; Liu, X., Wnt5a promotes epithelial-to-mesenchymal transition and metastasis in non-small-cell lung cancer. *Bioscience Reports* **2017,** 37, (6).

22. Giannakis, M.; Hodis, E.; Jasmine Mu, X.; Yamauchi, M.; Rosenbluh, J.; Cibulskis, K., . . . Garraway, L. A., RNF43 is frequently mutated in colorectal and endometrial cancers. *Nat Genet* **2014,** 46, (12), 1264-6.

23. Hopp, L.; Wirth, H.; Fasold, M.; Binder, H., Portraying the expression landscapes of cancer subtypes: A glioblastoma multiforme and prostate cancer case study. *Systems Biomedicine* **2013,** 1, (2), 99-121.

24. Loeffler-Wirth, H.; Kreuz, M.; Hopp, L.; Arakelyan, A.; Haake, A.; Cogliatti, S. B., . . . Binder, H., A modular transcriptome map of mature B cell lymphomas. *Genome Medicine* **2019,** 11, (1), 27.
